# Capturing Hu Antigen R Domain Closure Through Supervised Molecular Dynamics Simulations

**DOI:** 10.1002/cmdc.70523

**Published:** 2026-10-04

**Authors:** Chiara Cavastracci Strascia, Andrea Dodaro, Gianluca Novello, Silvia Menin, Mattia Sturlese, Veronica Salmaso, Stefano Moro

**Affiliations:** ^1^ Molecular Modeling Section (MMS), Department of Pharmaceutical and Pharmacological Sciences University of Padova Padova Italy

**Keywords:** conformational dynamics, domain closure, HuR (ELAVL1), structure‐based drug design, supervised molecular dynamics

## Abstract

Hu antigen R (HuR), an RNA‐binding protein implicated in cancer, inflammation, and neurodegenerative disorders, represents a challenging target for drug discovery due to its large solvent‐exposed RNA‐binding interface and pronounced conformational flexibility. HuR contains tandem RNA recognition motifs (RRM1 and RRM2) that undergo substantial rearrangements between an apo open state and an RNA‐bound closed conformation. Here, we investigated both states using molecular dynamics (MD) simulations. While the RNA‐bound complex maintained a stable closed architecture, the apo form displayed extensive interdomain motions, highlighting the intrinsic flexibility of HuR. Because conventional MD simulations failed to capture the open‐to‐closed transition, we developed a novel application of Supervised MD (SuMD), previously employed for intermolecular recognition processes, to characterize HuR intramolecular domain closure. Using a multistep supervision protocol based on selected interdomain residue pairs, SuMD reproduced a closed conformation resembling the experimental RNA‐bound structure and identified a plausible reclosure pathway. Per–residue interaction analyses revealed key contributions from the RRM1–RRM2 linker region, particularly Arg97, together with Arg136 and Arg147 of RRM2, consistent with mutagenesis data. These findings extend the applicability of SuMD to intramolecular conformational transitions, provide mechanistic insight into HuR domain closure, and offer a structural framework for identifying druggable conformational states for future HuR‐targeted drug discovery.

## Graphical abstract

HuR is a therapeutically relevant RNA‐binding protein that switches from an open apo form to a closed RNA‐bound one. Its atomistic detail is unknown. Here, Supervised Molecular Dynamics is applied for the first time to an intramolecular event: a multistep supervision of interdomain distances reproduces a plausible closure pathway and reveals transient putative pockets of potential interest for drug design.

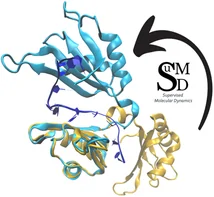

## 1 Introduction

Hu antigen R (HuR) is an RNA‐binding protein (RBP) that regulates posttranscriptional gene expression by interacting with adenylate‐ and uridylate‐rich elements (AREs) typically located within the 3′‐untranslated regions (UTRs) of target mRNAs. It belongs to the Hu/ELAVL (embryonic lethal abnormal vision‐like) family, which comprises four members: HuA/HuR, HuB, HuC, and HuD. Among these, HuR is ubiquitously expressed, whereas the other members are predominantly restricted to neuronal tissues [1, 2, 3].

Structurally, HuR is composed of three RNA recognition motifs (RRM1, RRM2, and RRM3), each consisting of approximately 80 amino acids and adopting the canonical β_1_α_1_β_2_β_3_α_2_β_4_ topology, characterized by a four‐stranded antiparallel β‐sheet packed against two α‐helices. Within each RRM, the central β‐strands (β_1_ and β_3_) contain the conserved ribonucleoprotein motifs RNP‐2 (β_1_) and RNP‐1 (β_3_), which are essential for nucleic acid binding [1, 4, 5]. Functionally, RRM1 and RRM2 are primarily responsible for RNA binding [1], whereas RRM3 contributes to the stabilization of the RNA–protein complex [6] and mediates interactions with the poly(A) tails of target mRNAs. Moreover, RRM3 is thought to be involved in the mRNA‐induced cooperative assembly of HuR oligomers [2].

RRM1 and RRM2 are connected by a short, flexible linker of approximately ten amino acids, while RRM2 and RRM3 are separated by a longer, disordered hinge region of about 60 amino acids. This region contains the HuR nucleocytoplasmic shuttling sequence, which enables HuR to translocate from the nucleus to the cytoplasm, thereby promoting mRNA stabilization and/or enhancing translational efficiency [1, 5, 7]. Under physiological conditions, HuR is predominantly localized in the nucleus, where it participates in pre‐mRNA splicing and the nuclear export of mature transcripts, although its nuclear functions remain only partially understood. Upon specific stimuli, including cellular stress and mitogenic signals, HuR undergoes nucleocytoplasmic shuttling and accumulates in the cytoplasm, where it exerts its major roles in mRNA stabilization and translational regulation [7].

Consistent with its regulatory role, HuR overexpression and its cytoplasmic accumulation have been implicated in tumorigenesis, as well as in neurodegenerative and inflammatory disorders, through the modulation of mRNAs encoding proteins involved in cell proliferation, survival, angiogenesis, invasion, pharmacoresistance, and metastasis [8]. Accordingly, HuR has emerged as a potential therapeutic target.

From a medicinal chemistry perspective, studies have largely focused on the RRM1–RRM2 tandem domains, which nonetheless pose significant challenges. First, the protein exhibits high conformational plasticity: superposition of the first domain of unbound (PDB ID: 4EGL) and bound (PDB ID: 4ED5) structures reveals an approximately 180° rotation of the second RRM domain. Furthermore, the apo conformation shows minimal interdomain contacts between RRM1 and RRM2, whereas the RNA‐bound conformation displays a limited number of interresidue interactions between the two domains [1]. In the RNA‐bound complex, the two domains adopt a closed arrangement, forming a positively charged cleft that accommodates RNA binding [1]. Accordingly, this conformation is commonly used in modeling studies. In particular, docking, virtual screening, molecular‐dynamics simulations, and free‐energy calculations have been used to identify and investigate the interdomain cleft between RRM1 and RRM2 as a key site for interfering with HuR‐RNA recognition [5, 9, 10, 11, 12, 13, 14, 15, 16]. In close synergy with biophysical techniques, these approaches have enabled the design of RNA‐mimetic inhibitors [11, 14, 16, 17] and the interpretation of experimental findings for identified ligands, providing atomistic insight into binding poses, key residue contacts, conformational effects, and structure–activity relationships [5, 13, 16]. However, HuR closed conformation lacks well‐defined binding pockets suitable for small molecules.

These features complicate the structure‐based identification of compounds capable of disrupting protein‐RNA interactions and acting as inhibitors of HuR. Understanding the mechanism of the conformational transition of the protein from the unbound to the RNA‐bound state can aid the identification of transient clefts on the protein surface that are potentially easier to target with small‐molecule ligands. For this reason, here, we present a detailed structural analysis of HuR aimed at characterizing its conformational landscape and identifying structural states that may be more suitable for small‐molecule binding. In particular, the application of Supervised Molecular Dynamics (SuMD) to simulate the closure pathway of HuR is here assessed.

## 2 Results and Discussion

### 2.1 MD Simulations of HuR in the Open and Closed Conformation

The RBP HuR is composed of three RRM motifs, with a linker of approximately ten amino acids connecting the two N‐terminal tandem RRM1 and RRM2 domains and a linker of approximately 60 amino acids connecting RRM2 and RRM3. No experimental structure of the complete protein has been reported so far, but structures of the isolated RRM1 [18, 19, 20] and RRM3 [4, 21] domains and of the tandem RRM1‐RRM2 portion are present in the Protein Data Bank (PDB).

In this work, we have focused on the two experimental structures available for the tandem RRM1‐RRM2 domains, which are primarily involved in RNA binding. Hereafter, the tandem RRM1‐RRM2 construct will be denoted as “HuR” in the text to simplify reading. The two X‐ray structures represent an apo (PDB ID: 4EGL, Figure 1A) and an RNA‐bound (PDB ID: 4ED5, Figure 1B) state of HuR, where the protein is bound to an 11‐nucleotide AU‐rich RNA sequence. The apo and the RNA‐bound conformations differ by a huge rotation of RRM1 relative to RRM2, as shown after overlapping the RRM1 domains (Figure 1C). The superposition highlights the conformational plasticity of the protein, with the apo form being characterized by an open conformation and the RNA‐bound form by a closed one.

**FIGURE 1:**
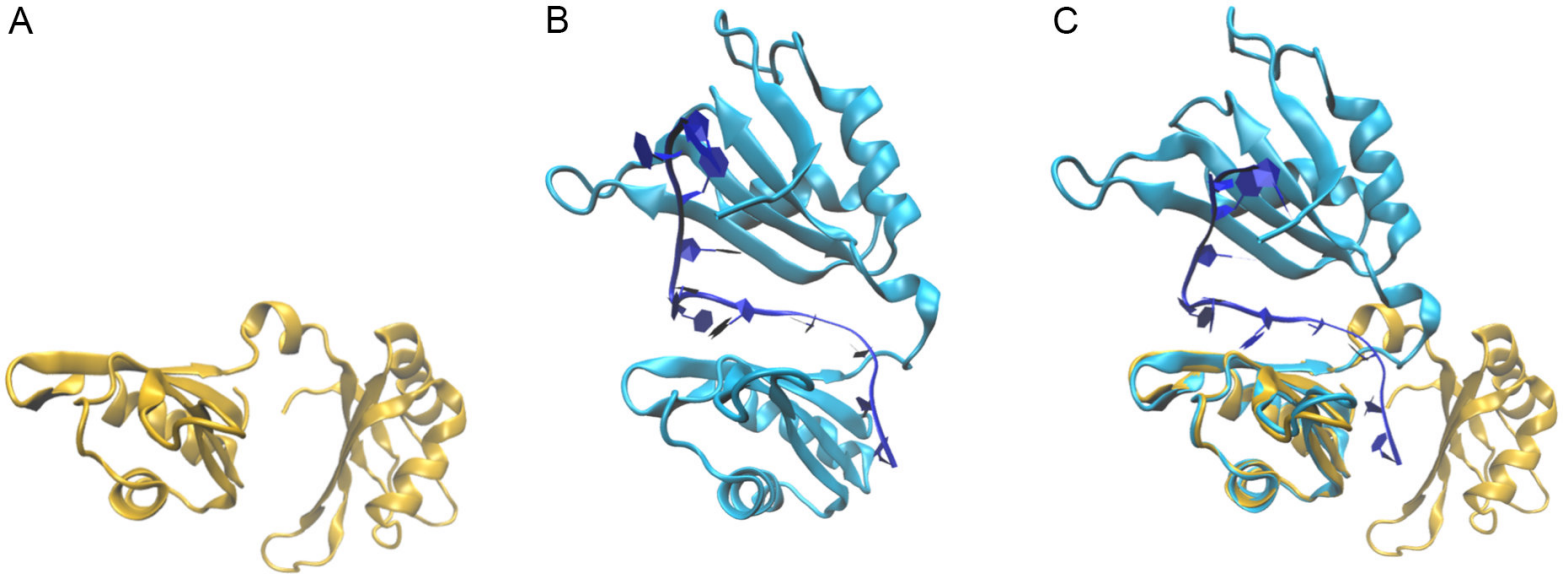
(A) Apo (PDB ID: 4EGL, yellow) and (B) RNA‐bound (PDB ID: 4ED5, protein in cyan, RNA in blue) states, and (C) their structural superimposition on the RRM1 domains of HuR.

The conformational stability of the protein was assessed by means of molecular dynamics (MD) simulations, and in particular, three replicates of 500 ns MD simulations were conducted both for the apo and RNA‐bound structures. All the trajectories were aligned on the backbone of RRM1, and the RMSD of RRM2 backbone atoms was computed using the initial structure as reference. RRM1 was defined as the Gly18‐to‐Ala96 range, and RRM2 as Ala106‐to‐Asn186. These boundaries were selected to ensure the inclusion of the secondary structure elements of the two RRM domains (i.e., extending one amino acid beyond the last/first β‐strand of RRM1/RRM2). As evident from RRM2 RMSD profiles, while the conformation of HuR appears stable in the RNA‐bound state, with an average interreplicate RRM2 RMSD value of 2.65 Å, it shows high flexibility in the apo state (average 33.00 Å RMSD), as reported in Figure 2A,B and Table 1. The Cα‐RMSF analysis reported in Figure 2D–F confirms this behavior: after alignment on the initial state RRM1 backbone, the Cα‐RMSF of RRM2 shows ubiquitously high values in case of the apo structure (with minimum and maximum RMSF values of 10.40–34.53 Å, 3.30–12.89, Å, 5.82–14.85 Å in the three replicates, respectively), while in the case of the RNA‐bound protein, the RMSF values drop (minimum and maximum RMSF values of 1.08–3.71 Å, 1.10–4.13 Å, 1.08–3.72 Å in the three replicates, respectively). The few residues with Cα‐RMSF around 4 Å include Thr142, Thr143, Pro173, Gly174, Ser175, and Ser176, which are located in highly flexible regions such as loops connecting β‐strands or linking a β‐strand to an α‐helix.

**FIGURE 2:**
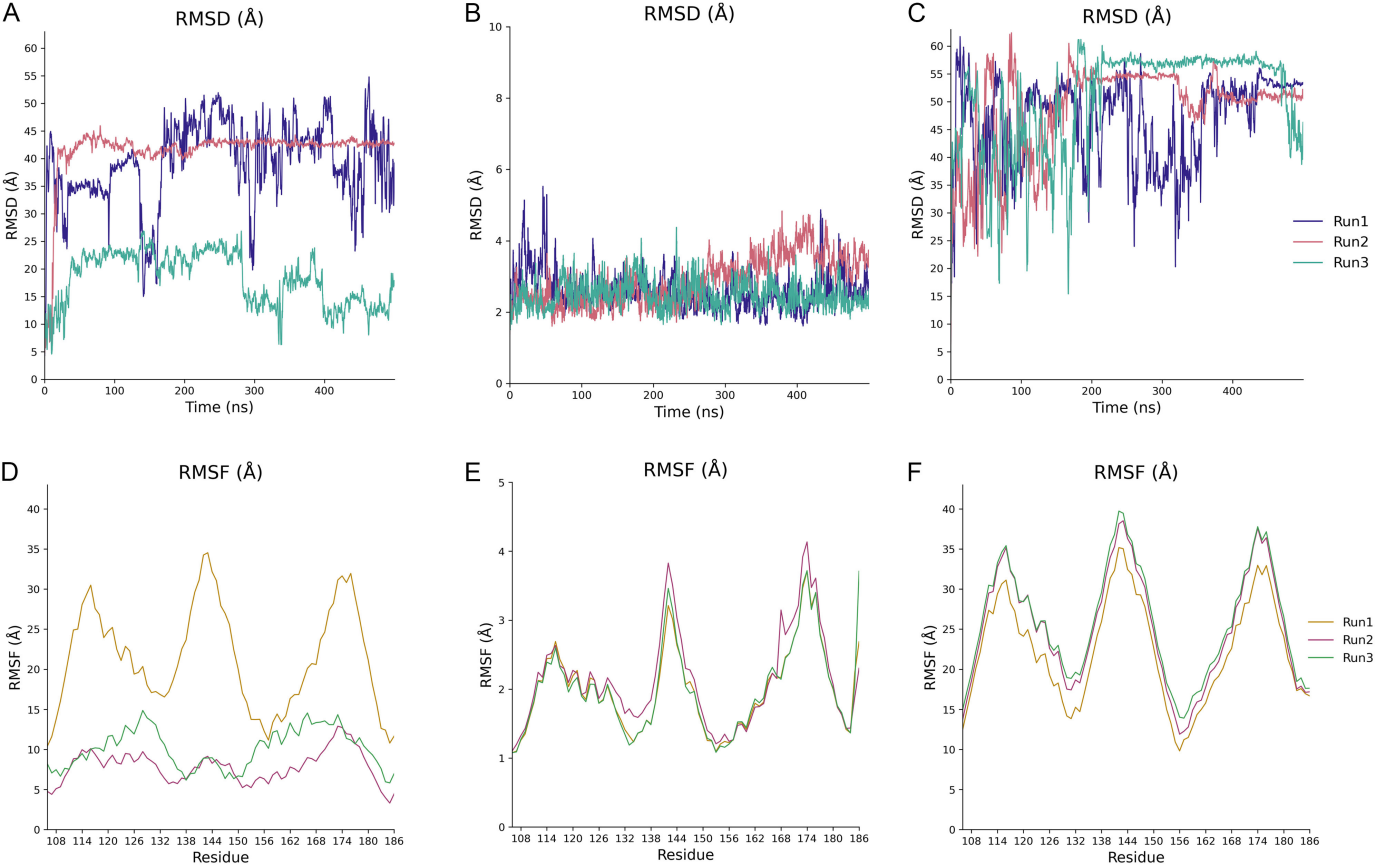
Backbone RMSD and Cα‐RMSF profiles of HuR RRM2 from three 500 ns MD replicates for the apo state (A and D, respectively), RNA‐bound state (B and E, respectively), and pseudo‐apo state (C and F, respectively), after alignment on the initial structure RRM1 backbone atoms. RMSD was computed using the relative initial HuR structure as reference (specifically, its RRM2 domain).

**TABLE 1:** Mean RRM2 backbone RMSD values for HuR using initial position as reference in the apo, crystal RNA‐bound and pseudo‐apo states across three independent MD replicates. For each condition, the table reports the average RMSD of each run and the overall interreplicate mean. RMSD was computed by aligning the trajectories on RRM1 backbone initial conformation, and computing RRM2 backbone RMSD. Standard deviations are also reported for each mean value.

State	Run 1 mean	Standard deviation	Run 2 mean	Standard deviation	Run 3 mean	Standard deviation	Interreplicate mean	Standard deviation (all data)
RRM2 RMSD apo	39.22 Å	7.59	41.49 Å	5.33	18.30 Å	4.93	33.00 Å	12.08 Å
RRM2 RMSD crystal	2.60 Å	0.74	2.88 Å	0.83	2.48 Å	0.66	2.65 Å	0.76 Å
RRM2 RMSD pseudo‐apo	46.68 Å	7.88	48.90 Å	8.00	50.70 Å	9.13	48.76 Å	8.52 Å

The high RMSD and Cα‐RMSF values observed for the apo system are due to the reciprocal movements of the tandem domains RRM1 and RRM2, while the internal topology of the two domains is maintained over time. Indeed, RRM1 and RRM2 were characterized by low RMSD and Cα‐RMSF when aligned with their respective backbone initial conformation. Specifically, the interreplicate average RMSD was 1.26 Å for RRM1 and 2.14 Å for RRM2 (Figure S1 and Table S1), with Cα‐RMSF falling below 2.5 Å for almost all residues (Figure S2A) and few exceptions located in the protein termini or loops (i.e., N‐terminal Gly18, Asn168, Gly169, His170, Lys171, Pro173, Gly174, Ser175, and C‐terminal Asn186).

Aiming to verify if the higher rigidity of the RNA‐bound structure is due to the presence of RNA or to the closed conformation of HuR, we conducted MD simulations of the protein starting from the closed state deprived of RNA (called “pseudo‐apo” hereinafter). High conformational rearrangements could be appreciated as well (average 48.76 Å RMSD, Figure 2C–F and Table 1), suggesting a stabilizing effect of RNA on HuR conformation. Moreover, we verified if the closed state of HuR was reached in the absence of RNA during the apo MD simulation: a closed crystal‐like conformation (lowest RMSD of 7.88 Å) was reached only transiently during one of the simulations (run1) but maintained for only a few frames (Figure S3).

As further proof of the stability of the RNA–HuR complex, the interaction energies between RNA and protein residues were monitored over time. The protein residues most frequently in contact with RNA (within a 4.5 Å radius) are reported in Figure 3, and they recall the pattern of interactions observed in the X‐ray structure of the complex. The most stabilizing interactions involve Lys89, Lys104, Arg136, Arg147, and Arg153, which are engaged in salt bridges with the phosphate groups. Phe151, Phe65, Tyr26, and Tyr109 are involved in π–π stacking interactions with U4, U10, U5, and U3, respectively. Ile23, Ile52, Leu61, Tyr63, Thr90, Ala96, Ser99, Lys92, Ile103, Asn107, Asn134, and Leu138 seem to be involved in hydrogen bonds with the nucleic acid. Asn25, Lys50, and Arg97 are involved both in hydrogen bonds and in salt bridges.

**FIGURE 3:**
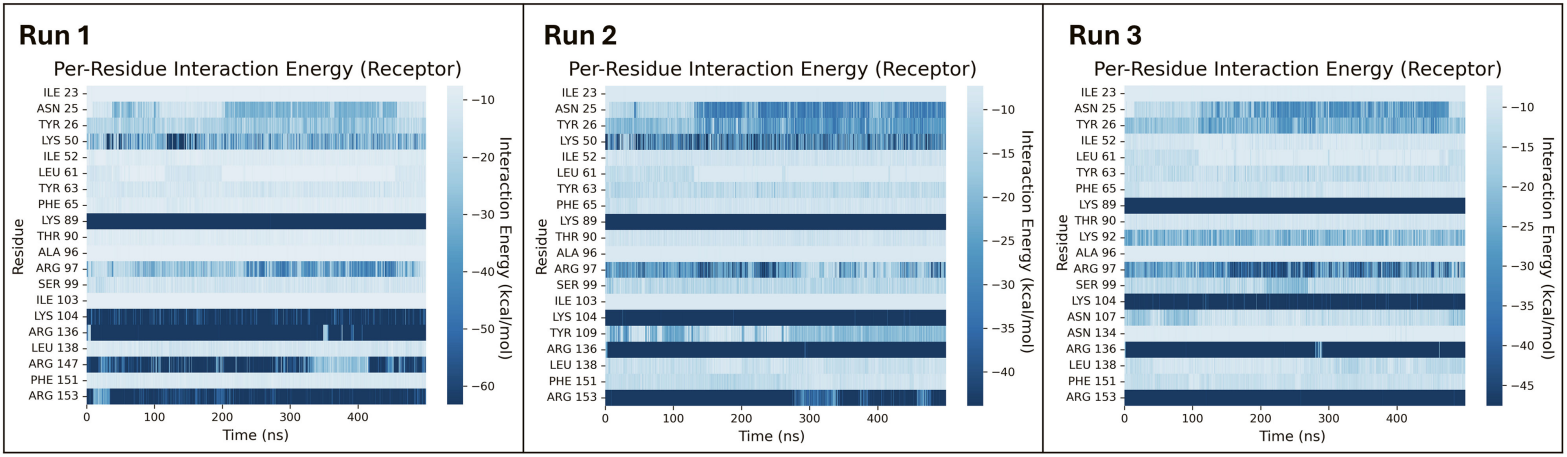
Perresidue interaction energy profiles between RNA and HuR during the MD simulations across the three replicates, highlighting the twenty protein residues most frequently in contact with RNA (within a 4.5 Å radius).

### 2.2 Simulations of HuR Conformational Transition

As mentioned above, the RNA binding cleft on HuR is defined by the interface between RRM1, RRM2, and the linker. Considering the high flexibility of HuR in the unbound state and its high stability in the RNA‐bound one and aiming to further assess the stabilization effect of RNA and its impact on the conformational rearrangement of HuR, we investigated the possibility to simulate a reclosure pathway of HuR starting from the apo state in the presence of RNA positioned on the RRM1 domain.

The RNA strand was retrieved from the RNA‐bound closed structure and was placed on the RRM1 RNA‐binding surface. In particular, the RRM1 domains of the RNA‐bound/closed structure (PDB ID: 4ED5) and of the apo/open structure were aligned, and the RNA coordinates were transferred to the apo structure (Figure 4). The choice of RRM1 over RRM2 for RNA placement was supported by experimental evidence reporting that RRM1 has the highest affinity for RNA [1] as compared to RRM2 and is involved earlier in RNA recognition [1].

**FIGURE 4:**
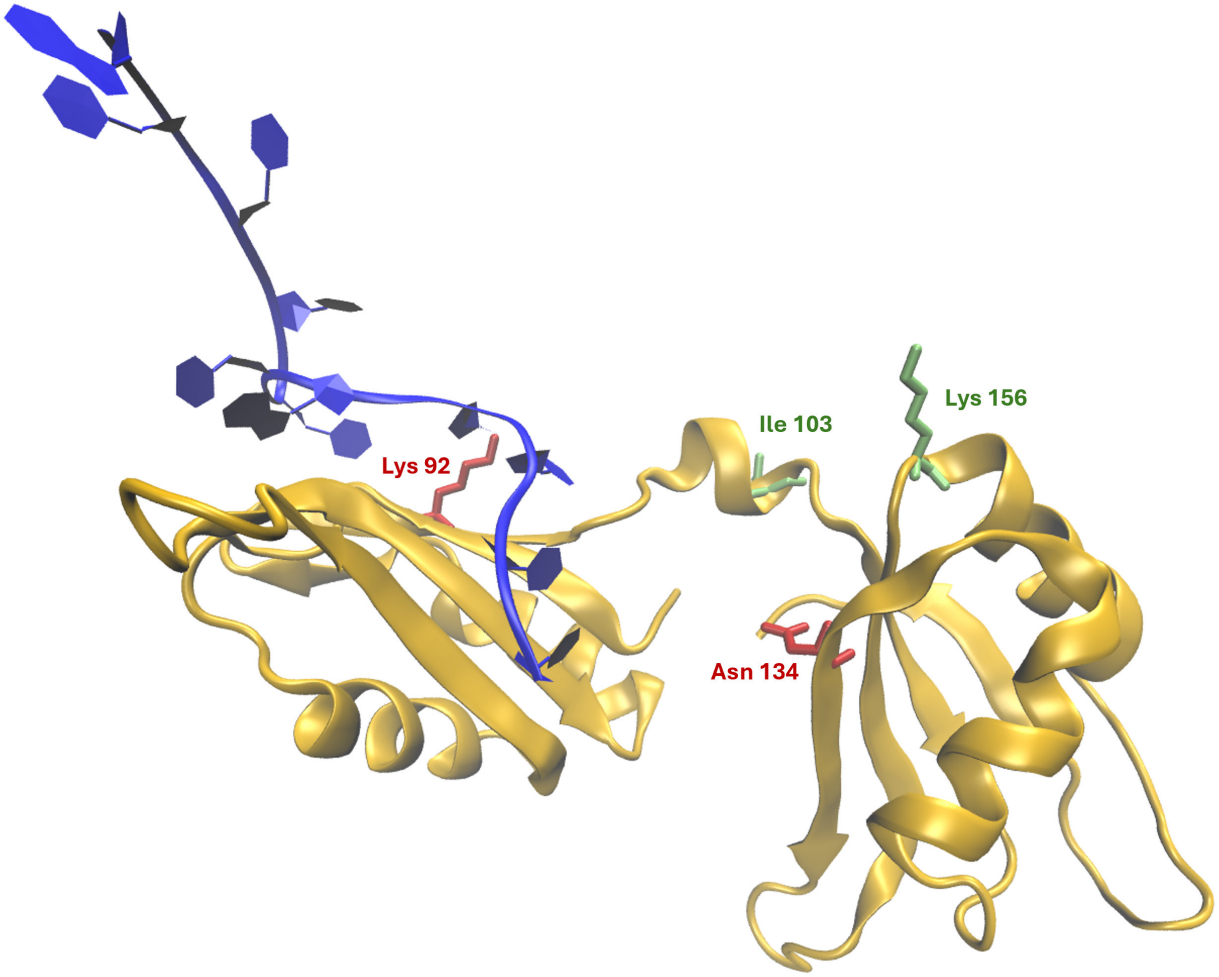
Artificial HuR–RNA open complex generated by superposing the RRM1 domain of the RNA‐bound closed structure (PDB ID: 4ED5) onto the RRM1 domain of the apo HuR structure (PDB ID: 4EGL). RNA extracted from the closed structure is shown in blue, whereas the apo HuR structure is shown in yellow. Moreover, the residue pairs used for SuMD supervision are shown. The red residues represent the monitored residues in Case 1 Supervision Step‐1, whereas the lime residues represent the monitored residues in Case 2 Supervision Step‐1.

Three 500 ns runs of classical MD simulations were conducted starting from this construct, but despite the movements of RRM2 relative to RRM1, none of the simulations resulted in the reproduction of the experimental conformation of the closed RNA‐bound state of HuR, with an interreplicate average RMSD of 47.12 Å from the X‐ray closed conformation (Figure S4).

The simulation time required to observe a reclosure pathway leading to a crystal‐like topology might require more than 500 ns. For this reason, we employed SuMD, a tool previously developed in our laboratory to study ligand‐target binding pathways and recently applied to simulate the recognition of proteins with intrinsically disordered regions to nucleic acids [22]. SuMD employs multiple subsequent short classical molecular dynamics simulations (SuMD steps) and monitors the approach of two molecules or portions of molecules during each step, focusing sampling in the direction of productive events. In particular, the distance between the centers of mass of two selected molecules (or portions of molecules) is computed at regular intervals during each SuMD step, and the slope of the line fitting the distances over time is used to discriminate a productive SuMD step. When the slope is lower than 0, the SuMD step is leading the two monitored partners to approach each other; the step is therefore considered productive, is stored, and a new SuMD step is started from the final coordinates of the previous one. Conversely, if the slope is higher than 0, the SuMD step is discarded and restarted by reassigning velocities. This algorithm was initially developed to simulate ligand‐target recognition, where the distance between the center of mass of the ligand and the target selected residues was monitored. It proved to be effective in reproducing the binding modes of ligand‐target complexes in good agreement with experimental structural data, with examples of targets spanning from GPCRs, globular proteins, nucleic acids, and ligands ranging from small molecule to peptides and nucleic acids. The great advantage of SuMD is the reduction of the time scale required to observe a rare event such as binding, from microsecond to nanosecond timescales [23, 24, 25, 26, 27, 28]. Here, we assessed whether SuMD could be applied to simulate the reclosure pathway of HuR, leading to what is, to our knowledge, the first application of SuMD to an intramolecular conformational transition. In particular, the recognition pathway of the RRM2 domain toward the RRM1‐bound RNA was simulated.

The first SuMD attempts involved the supervision of the centers of mass of the RNA‐binding interface of RRM1 and RRM2, defined as the RRM1 and RRM2 regions falling within a 4.5 Å radius from the nucleic acid in the closed X‐ray structure of HuR. RRM1 residues involved Asn21, Ile23, Asn25, Tyr26, Gln29, Glu47, Ser48, Lys50, Ile52, Lys55, His59, Ser60, Leu61, Gly62, Tyr63, Phe65, Asn67, Lys89, Thr90, Ile91, Lys92, Ser94, Ala96, and RRM2 residues involved Arg97, Pro98, Ser99, Ser100, Ile103, Lys104, Asp105, Asn107, Tyr109, Ile132, Ile133, Arg136, Leu138, Arg147, Val149, Phe151, Arg153, Lys182, Phe183, Ala184, Ala185, Asn186, NME187. Four SuMD runs were performed, but none of them succeeded in reproducing the experimental closed RNA‐bound conformation of HuR. Indeed, the RMSD between the experimental closed conformation of RRM2 and the conformation reached at the end of each SuMD simulation never dropped below 44 Å (data not shown). The supervision of the distance between the centers of mass of the two RRM interfaces seemed insufficient to focus sampling on the conformational rearrangement required to obtain an RNA‐bound crystal‐like closed RRM1‐RRM2 topology. For this reason, a different SuMD supervision was attempted, with the idea of focusing the SuMD supervision on pairs of residues better representing the two extreme open versus closed conformations of HuR.

In this context, pairs of residues with markedly different reciprocal distances in the open versus closed conformation of HuR should undergo large movements during the HuR reclosure mechanism and could thus be used to monitor SuMD simulations. To avoid choosing residues that are located at a greater distance in the open state but that are not very close in the closed state, pairs of residues with marked proximity in the HuR‐closed state were chosen.

We thus computed the pairwise distance matrices of the Cα carbon atoms in the open and closed HuR conformations and then the difference between the two matrices (open minus closed) to obtain the pairwise Δ‐distance of each residue pair between the open and closed states. The residue pairs were ranked on the basis of the Δ‐distance value (the higher, the better) and on the basis of their distance in the closed form (the closer, the better). The three top‐ranked residue pairs were Leu61‐Asn186, Lys92‐Asn134, and Ile103‐Lys156 (Table S2).

The Leu61‐Asn186 pair was not considered because it involves residue Asn186, which is an artificially capped C‐terminus located at the truncated extreme of the protein construct employed in this work. Indeed, as previously mentioned, the protein here is the tandem RRM1‐RRM2 portion of HuR, lacking a further physiological extension (60‐residue hinge region and RRM3 motif at the C‐terminal extreme). Thus, we assumed that the mobility of Asn186 might differ from that in the full HuR protein, making it suboptimal for supervision. The Lys92‐Asn134 and Ile103‐Lys156 residue pairs were thus considered for supervision.

Residues Lys92 (RRM1) and Asn134 (RRM2) are located at a distance of 26.45 Å in the open HuR conformation and of 10.99 Å in the closed conformation (resulting in Δ‐distance of 15.46 Å). While residues Ile103 (linker) and Lys156 (RRM2) are 13.01 Å apart in the open form and 4.46 Å apart in the closed one (resulting in Δ‐distance of 8.56 Å).

The distances between the centers of mass of the two residue pairs were thus used to monitor different sets of SuMD simulations. Starting from the same initial apo state with RNA placed on RRM1, 4 runs of SuMD simulations were conducted by monitoring the Lys92‐Asn134 centers of mass distance (Case 1), and 4 separate runs were conducted by monitoring the Ile103‐Lys156 centers of mass distance (Case 2). The positions of the selected residue pairs in the apo state are shown in Figure 4. In Case 1, Lys92 and Asn134 are approximately 26 Å apart at the beginning of the simulation, and they approach each other during all the simulations down to an approximate distance of 19 Å in run1 and run3 (Table 2, marked as *). However, in none of the runs did HuR reach a crystal‐like pose, with an RRM2 RMSD higher than 40 Å in all cases.

**TABLE 2:** Multistep Unproductive SuMD. RRM2 (backbone) RMSD with respect to the X‐ray closed structure (PDB ID: 4ED5) after alignment on RRM1, HuR–RNA interaction energy and supervised distance, all evaluated on the final frame of each simulated SuMD step of the Multistep Unproductive protocol. The best run of each Supervision Step is marked with an asterisk (*).

Multi‐step Unproductive SuMD	SuMD run	Last frame RMSD, Å	Last frame energy, kcal/mol	Last frame center of mass distance, Å
First supervision step: Lys92‐Asn134	Run1 *	44.90	−625.87	19.40
	Run2	47.13	−403.02	22.58
	Run3	46.95	−506.26	19.37
	Run4	47.70	−479.66	22.71
Second supervision step: Ile103‐Lys156	Run1 *	45.37	−730.46	9.14
	Run2	43.69	−688.14	13.04
	Run3	45.12	−618.08	13.84
	Run4	43.76	−710.57	15.21

In Case 2, the distance between Ile103 and Lys156, which starts at approximately 13 Å in the initial state, diminished in all SuMD runs, reaching a final distance of 5.10 Å in run 1 (Table 3, marked as **), similar to the crystallographic closed‐state value. The RMSD value of RRM2 between the final state of run1 and the closed crystallographic HuR conformation was 14.57 Å, markedly below all the other runs (Table 3 and Figure S5A). The interaction energy between HuR and RNA during the simulation was thus computed (Figure S6) and compared with the other runs, and it appeared that the final state reached in run1 is more stable than the others (last frame interaction energy of −560.60 kcal/mol for run1, while higher than −500 kcal/mol in the other runs, as reported in Table 3). The SuMD approach was still not sufficient to sample the full reclosure pathway of HuR to the crystal‐like RNA‐bound state, but the conformation obtained in run1 seemed to anticipate it.

**TABLE 3:** Multistep Productive SuMD. RRM2 (backbone) RMSD with respect to the X‐ray closed structure (PDB ID: 4ED5) after alignment on RRM1, HuR–RNA interaction energy and supervised distance, all evaluated on the final frame of each simulated SuMD step of the Multistep Productive protocol. The best run of each Supervision Step is marked with two asterisks (**).

Multistep Productive SuMD	SuMD run	Last frame RMSD, Å	Last frame energy, kcal/mol	Last frame center of mass distance, Å
First supervision step: Ile103‐Lys156	Run1 **	14.57	−560.60	5.10
	Run2	51.19	−484.67	9.67
	Run3	51.90	−343.40	8.10
	Run4	52.60	−406.05	13.74
Second supervision step: Lys92‐Asn134	Run1 **	9.04	−748.17	10.17
	Run2	17.30	−504.46	6.56
	Run3	12.03	−630.90	23.34
	Run4	14.02	−525.82	23.01

Recently, a novel application of SuMD was employed by G. Novello et al. [22], where the tool was used in a multistep approach to simulate the recognition between nucleic acids and proteins containing intrinsically disordered regions. In particular, the multistep approach involved a first SuMD run with supervision of selected residues of the protein unfolded region toward the nucleic acid, followed by a second run where the supervision involved residues of the protein ordered region and the nucleic acid. Therefore, here, we employed a similar multistep approach to simulate the HuR reclosure pathway, starting from the final states collected in Case 1 and Case 2 (described above as Supervision Step‐1) and conducting a second SuMD supervision by monitoring the other residue pair (Supervision Step‐2).

In particular, we first exploited the supervision of the Ile103‐Lys156 distance as Supervision Step‐1 (Case 2 described above), and the most productive final state of run1 was selected as the starting point for Supervision Step‐2, where the distance between Lys92 and Asn134 was monitored. Four SuMD runs were also collected in the context of Supervision Step 2. Among them, run1 succeeded in reproducing a nearly crystal‐like conformation of closed RNA‐bound HuR, with an RMSD of 9.04 Å for the RRM2 backbone. Run2 reached the lowest Lys92‐Asn134 distance (6.56 Å), but run1 (with a final Lys92‐Asn134 distance of 10.17 Å) was associated with the best interaction energy profile (Figure S6B) and better reproduced the crystal‐like RNA‐bound closed topology of HuR (Figure 5). The RMSD value of 9.04 Å between the RRM2 final state and the X‐ray‐closed structure may appear high, but the overall relative orientation of RRM1 and RRM2 closely reproduces the experimentally closed topology. It should be considered that by forcing the alignment on RRM1, the geometrical difference of RRM2 is exacerbated, with higher per‐residue RMSD values for Arg115 (14.10 Å), Thr116 (14.99 Å), Thr142 (13.14 Å), Thr143 (13.76 Å), Leu145 (13.39 Å), Pro173 (14.99 Å), Gly174 (15.73 Å), Ser175 (14.69 Å), and Ser176 (15.13 Å). When aligning on the entire backbone and consequently computing RMSD over the whole backbone, the RMSD drops drastically to 4.33 Å. In this case, the highest per‐residue RMSD, excluding the terminal residues Gly18 (12.24 Å) and Asn186 (11.50 Å), is associated with Ala57 (8.05 Å), while all other residues remain below 8 Å. Moreover, when each RRM domain is aligned onto itself, the RMSD analysis yields a global RMSD of 1.19 Å for RRM1, with per‐residue values below 3 Å (except for the terminal Gly18 at 6.05 Å) and a global RMSD of 1.38 Å for RRM2, with per‐residue values below 3.05 Å (except for the terminal Asn186 at 5.18 Å), still highlighting the internal geometrical stability of each RRM domain. All the mentioned per‐residue RMSD profiles are reported in Figure S7.

**FIGURE 5:**
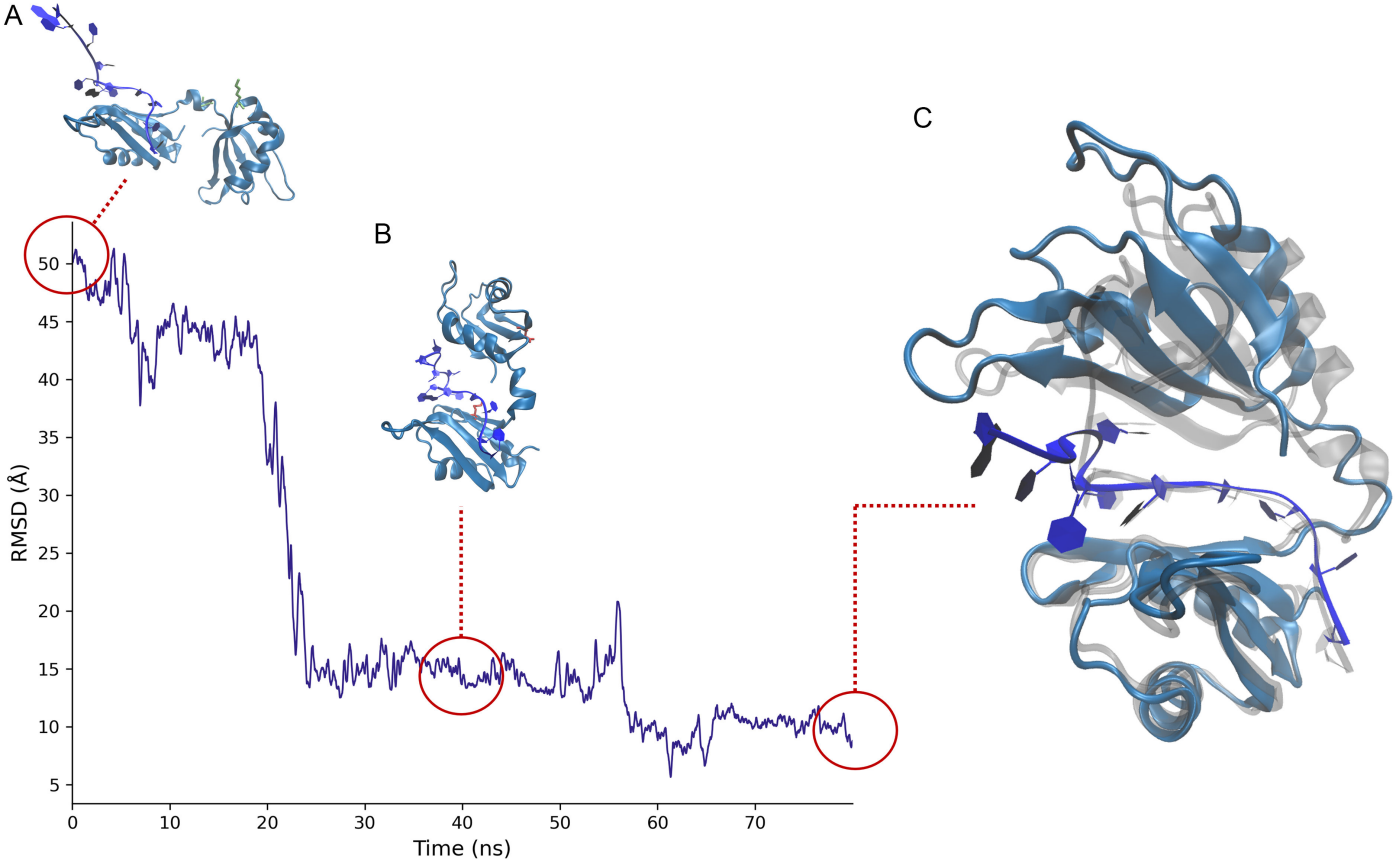
Representative SuMD productive trajectory showing the RRM2 RMSD profile over time versus the closed X‐ray structure (left). Three key frames are highlighted: the starting point (A), an intermediate structure at the end of the first supervision phase (B), and the final frame at the end of the second supervision phase (C). The starting position of residue pairs used for the supervision are shown in A (Ile103 and Lys156 colored in lime) and in B (Lys92 and Asn134 colored in red). On the right, the final SuMD frame (protein: light blue, RNA: dark blue) is superimposed on the X‐ray reference structure (gray, transparent) aligning on RRM1, and this illustrates the close agreement between the simulated binding pose and the crystallographic conformation (C).

The persistence of the final state was probed over 100 ns of classical MD simulation, and during this single unbiased trajectory, the SuMD‐derived state persisted without undergoing a major reopening, although the RRM2 RMSD did not decrease further toward the crystallographic reference (Figure S8). This calculation therefore provides a limited short‐timescale persistence assessment rather than evidence of converged long‐term stability.

The reversed multistep strategy was also assessed, but it was not productive. In this case, during Supervision Step‐1, the distance between Lys92 and Asn134 was monitored (see Case 1 above), and then, starting from the final state of run1, selected on the basis of the best protein–RNA interaction energy, four SuMD runs of Supervision Step‐2 were conducted by monitoring the distance between Ile103 and Lys156. All the runs led to a final conformation of the complex, in which RRM2 of HuR differs by more than 40 Å RMSD from the closed crystallographic state. A panel collecting the images of the last frame of all SuMD trajectories is shown in Figure S9 (Productive SuMD) and Figure S10 (Unproductive SuMD).

This suggests that monitoring the distance between residues belonging to the linker and to an RRM domain first and then monitoring the distance between residues located on the two different RRM domains is more effective in driving HuR reclosure around RNA into a near‐experimental RNA‐bound conformation. The major source of flexibility of the protein is indeed the linker, so it is more efficient to focus the conformational search of the linker‐accessible space in the direction of productive events. The identified pathway should be regarded as one plausible route among potentially multiple closure mechanisms.

To gain mechanistic insight into the reclosure process, we performed a per‐residue analysis of the interaction energy along the complete reclosure trajectory collected in the MultiStep Productive SuMD (combining Run1 of Supervision Step‐1 and Run1 of Supervision Step‐2), defining the linker‐RRM2 region as the receptor and the nucleic acid as the ligand. Notably, this analysis recovered several residues previously reported in the literature [1] as key residues for RNA recognition. Wang et al. [1] carried out alanine‐scanning mutagenesis of residues predicted to be essential for recognition and identified Arg97, Arg136, and Arg147 as the most critical, with the Arg97 mutation producing the largest reduction in binding. Consistently, our analysis of the 25 residues (Figure 6) most frequently contacting the nucleic acid confirmed the prominent role of Arg97, located in the linker, throughout the reclosure process as well as the engagement of Arg136 and Arg147 in its later stages. In addition, further linker residues such as Pro98, Ser99, and Val102 displayed substantial energetic contributions, reinforcing the central role of the linker in driving the conformational transition.

**FIGURE 6:**
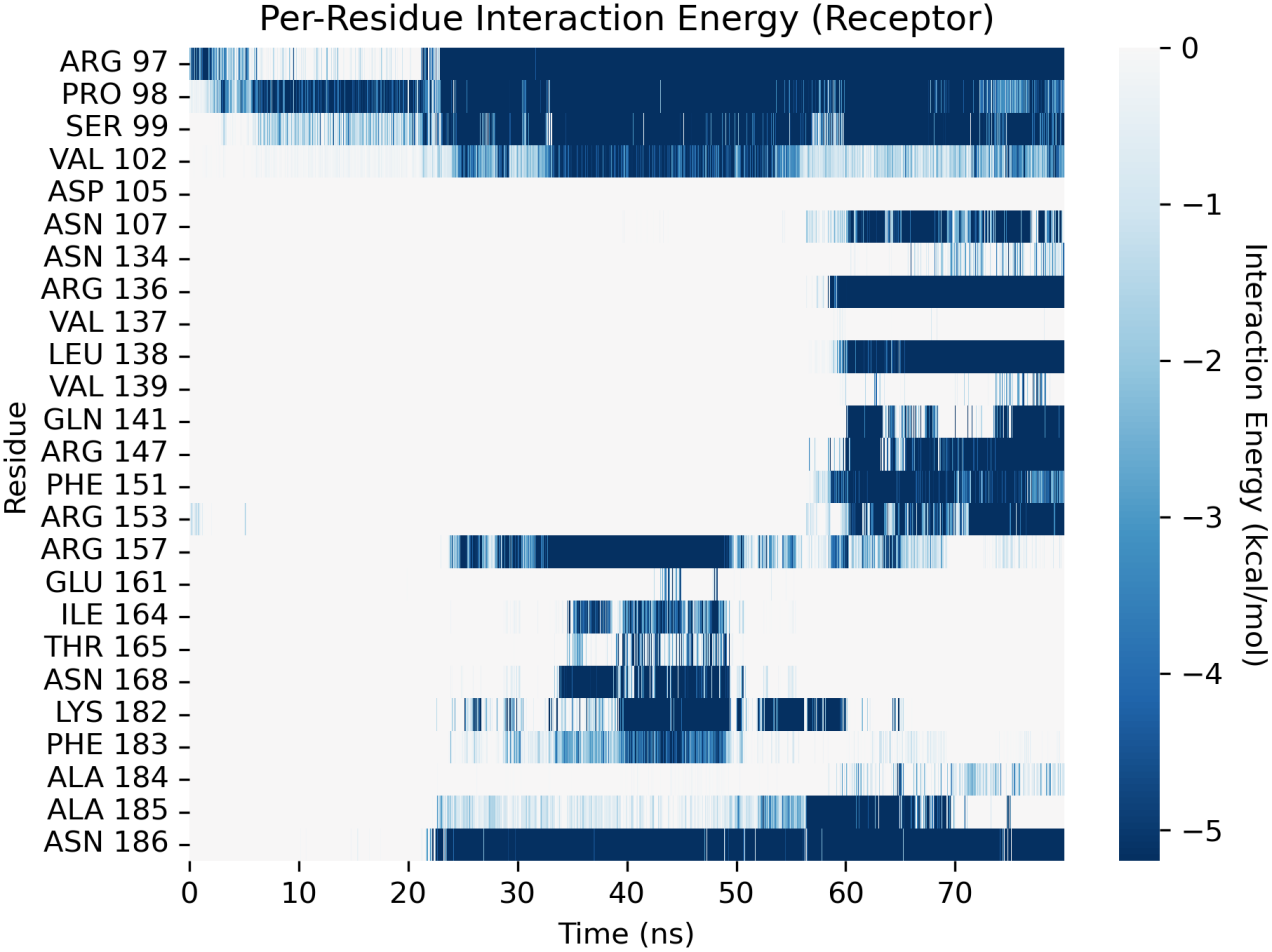
Per–residue interaction energy profile of HuR toward the nucleic acid along the trajectory, with the color scale representing the magnitude of the interaction energy in kcal/mol: lighter shades indicate weaker interactions, while darker blue tones correspond to stronger interactions between RNA and protein residues. Only the 25 most frequently contacting residues are shown.

Examining the individual contacts in greater detail, Phe151 established a stacking interaction with U4, while Phe183 formed a hydrogen bond with U2. Asn186 appeared to hydrogen‐bond with U8, and Lys182 formed hydrogen bond with U4 and a salt bridge with the phosphate groups of U3 and U5. Arg157 interacted with U9, and Arg153 formed salt‐bridge interactions with U8 and U9, with additional hydrogen bonds to surrounding nucleotides. Asn107 hydrogen‐bonded to U8. Arg136 formed a salt bridge with the phosphate of U5 and, in the final part of the trajectory, a hydrogen bond with U4, whereas Leu138 hydrogen‐bonded to U3, U4, and U5. Arg147 established a salt bridge with the phosphate of U2 and, in the last part of simulation, an additional contact with the phosphate of U3, while Gln141 hydrogen‐bonded to A1.

The behavior of Arg97 was particularly interesting: at the beginning of the simulation, its side chain pointed outward, away from the binding region, but following the rotation of the linker, it reoriented toward the interior of the newly formed cleft, interacting with nucleotides U9 and U10 and forming a salt bridge with the nearby phosphate groups.

Video S1 illustrates the SuMD reclosure process together with the protein–nucleic acid interaction energy and RRM2 RMSD profile along time. The video highlights the progressive movement of RRM2 toward the nucleic acid during reclosure. As the system approaches the closed topology, the interaction energy values become progressively more favorable, as indicated by the blue color scale, in which darker blue corresponds to more negative energy values.

Along the productive SuMD reclosure trajectory, a preliminary analysis was performed to identify transient putative pockets on the HuR–RNA complex surface. The trajectory was clustered using TTClust [29] with default settings, including auto‐clustering and Ward’s linkage method. Frames were aligned on the RRM1 domain, whereas the backbone RMSD of the full‐protein was used to define the number of clusters. This procedure yielded three representative structures, accounting for 27.56%, 44.98%, and 27.46% of the analyzed frames, respectively. These fractions refer to the frames of the selected concatenated SuMD trajectory and are not thermodynamic populations.

All representative structures were analyzed with Fpocket [30, 31] for pocket detection while retaining the nucleic acid in the system. In the first representative structure, eight pockets were identified, although only one displayed a druggability score above 0.5 (pocket 2: 0.521). The second structure contained sixteen pockets, none of which exceeded this threshold (the highest value is 0.207 for pocket 6). Finally, sixteen pockets were detected in the third structure, including one putative pocket with a high fpocket druggability score (0.898). It should be stressed that the fpocket druggability score is a geometry‐ and physicochemistry‐based estimator trained on protein binding sites; a high score is therefore an indication that a cavity deserves further assessment, not a demonstration that it is genuinely druggable, and this caveat is particularly relevant at a protein–nucleic acid interface such as the one examined here.

The druggable pocket (Figure 7A) identified in the first structure is delineated by Gly18, Arg19, Asn21, Tyr95, Ala96, Arg97, Pro98, Ala184, Ala185, Asn186, and nucleotides U9 and U10 and forms upon rotation of the interdomain linker. Residues Asn21, Ala96, Arg97, Pro98, Ala184, Ala185, and Asn186 are shared with those located within a 4.5 Å radius of the nucleic acid in the closed conformation. This set includes linker residues Arg97 and Pro98, which were found to be crucial for the rotation of RRM2 required to enable reclosure. In addition, residues Ala184, Ala185, and Asn186 emerge as important contributors during the final stage of the process.

**FIGURE 7:**
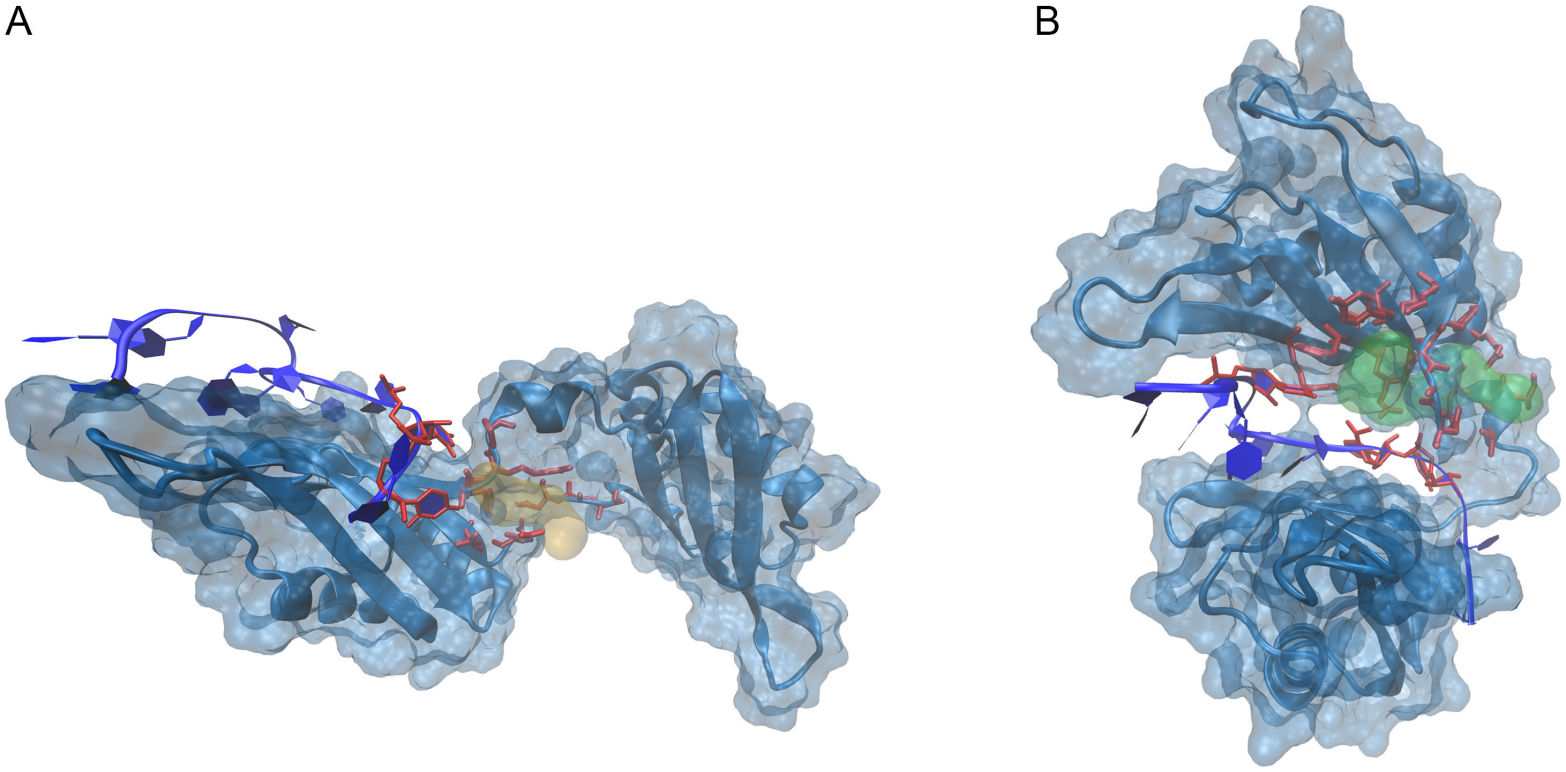
Transient pockets identified along the productive SuMD reclosure trajectory. (A) Pocket detected in cluster 1, shown in orange. (B) Pocket detected in cluster 3, shown in green. In both panels, the protein is displayed in light blue and the nucleic acid in dark blue, whereas residues delimiting each pocket are highlighted in red.

However, this pocket is substantially solvent‐exposed. In contrast, the pocket detected in the third representative structure is particularly noteworthy because it emerges as the protein approaches the closed conformation. Importantly, Fpocket did not identify an equivalent pocket in either the pseudo‐apo structure or the RNA‐bound complex. This cavity (Figure 7B) is defined by Val102, Ile103, Asp105, Asn107, Leu108, Tyr109, Arg136, Phe151, Ile152, Arg153, Arg157, Lys182, Phe183, Ala184, Ala185, Asn186, and nucleotides U4, U8, and U9, involving a larger set of residues than the pocket in the first structure. Moreover, it is less solvent‐exposed, suggesting a more favorable environment for ligand accommodation. All residues forming the pocket are also found within 4.5 Å of the nucleic acid in the X‐ray‐closed conformation, with the exception of Val102, Asn107, Ile152, and Arg157.

Although preliminary, these results indicate that the reclosure pathway may transiently expose potentially druggable sites that are not detectable in the endpoint conformations. These pockets will therefore be considered in future structural and ligandability assessments.

## 3 Conclusions

To investigate the conformational dynamics of HuR, in this work, we first performed molecular dynamics (MD) simulations on three different conformations of the protein: the apo form (PDB ID: 4EGL), the RNA‐bound form (PDB ID: 4ED5), and the pseudo‐apo conformation (protein starting from the closed‐state deprived of RNA). The results showed that only the crystallographic complex retained a stable closed conformation throughout the trajectories, suggesting that RNA binding plays a crucial role in stabilizing the closed state of the protein.

Based on these observations, we investigated the possibility of reproduce the closure process of the protein. An initial attempt was carried out by performing classical MD simulations of the apo structure after superposition of the RNA on RRM1; however, this strategy failed to promote a productive closure, prompting the application of SuMD.

The first SuMD approach was based on a single‐step supervision of the distance between the centers of mass of the RNA‐binding interface of RRM1 and RRM2, defined as the RRM1 and RRM2 regions falling within a 4.5 Å radius from the nucleic acid in the closed X‐ray structure of HuR. These simulations failed to reproduce the crystal‐like structure of the RNA‐bound closed state. The single supervision of the distance between selected pairs of residues was likewise not effective.

Inspired by the novel application of SuMD [22], where the tool was used in a multistep approach, we therefore implemented a similar protocol by selecting two pairs of residues better representing the two extreme open versus closed conformations of HuR.

Two distinct multistep protocols were designed. In the first case, supervision was initially applied to residues belonging to the linker region and RRM2, followed by residues located in the two structured RRM domains. In the second case, the supervision involved the same residues as in case 1 but in reverse order, first using the residue pair from RRM1 and RRM2, followed by a second step monitoring residues from the linker and RRM2.

The outcomes revealed a marked difference between the two protocols. Specifically, the first protocol successfully yielded a crystal‐like RNA‐bound closed conformation (RMSD ≈ 9 Å), which persisted without major reopening during a subsequent single 100 ns unbiased MD simulation. By contrast, the second protocol did not produce a productive reclosure event. These findings strongly suggest that linker rotation represents a key mechanistic prerequisite for driving the protein toward its closed conformation.

Furthermore, the analysis of interactions emerging between the linker‐RRM2 region and the nucleic acid throughout the trajectory of the crystal‐like form highlighted the involvement of residues previously reported in the literature as essential for nucleic acid recognition. Taken together, these findings indicate that SuMD identified a mechanistically plausible hierarchy of conformational events governing HuR closure.

Moreover, the pool of the collected trajectories also revealed novel conformational states accessible to the protein, which may represent valuable structural templates for future computer‐aided drug design studies and for the identification of transiently druggable conformational states.

This manuscript provides the first application of SuMD in supervising an intramolecular motion, further extending the applicability spectrum of the technique. The current limitation of the technique is the lack of a validated metric discriminating simulations ended into near‐crystallographic structure from decoys. We have observed that RNA–protein interaction energy in the final state of simulations is more favorable than in unproductive ones, but an absolute energetic threshold might not exist or differ among systems. SuMD simulations are intrinsically stochastic due to the random assignment of initial velocities and the stochastic velocity rescaling introduced by the Langevin thermostat. As a result, even small differences in the initial velocity distribution can propagate into markedly different trajectories, altering the exact sequence and timing of side‐chain contact formation. This explains why, among multiple replicas, only one yielded a productive closure pathway and underscores the difficulty of predicting *a priori* how many replicas are required to reliably capture such rare events. Therefore, the transition from open to closed states for systems lacking experimental structures could be prospectively simulated, but the issue arises about how many replicates to conduct before trusting the resulting pathway and final state as near‐native. Still, the prospective application of SuMD when structural information is lacking is possible and will generate initial hypothetical model structures, whose robustness should be strengthened by means of experimental data.

## 4 Materials and Methods

### 4.1 Hardware Overview

Structure preparation, simulation setup, and trajectory analysis were performed on a Linux workstation equipped with a 24‐core Intel Core i9‐14900K processor (6.0 GHz). Molecular dynamics simulations were performed on a cluster composed of 30 NVIDIA GPUs (GTX 980 and RTX5090).

### 4.2 Structure Preparation

All the basic molecular modeling operations were executed within the Molecular Operating Environment (MOE) [32] suite (version 2024.06). Each simulated system (apo, pseudo‐apo, RNA‐bound complex, and suMD complex) was prepared in the same way, starting from the three‐dimensional coordinates of the two structures deposited on the PDB (ID: 4EGL and 4ED5). Initially, the protein sequence downloaded from the PDB was compared with the complete FASTA sequence of the same protein and corrected where necessary. The structures were preprocessed through the “Structure Preparation” tool to assign the highest occupancy conformation to the residues with alternate states, build unresolved atoms and residues, and cap the N and C terminals. More specifically, Ala185 and Asn186 in the apo structure and Asn186 in the RNA‐bound structure were built to complete the second domain, and then, Asn186 was capped. Even the first two and the last bases of the RNA were rebuilt because they were not completely solved. Moreover, the hydrogens were added by considering the most probable protonation state at pH = 7.40 and temperature = 300 K, exploiting the “Protonate3D” tool. After that, the added hydrogens were minimized under the Amber:EHT force field implemented in MOE. At the end of the process, water molecules were removed. In both crystal structures, the modeled construct corresponds to the tandem RRM1–RRM2 region, spanning residues Gly18–Asn186 of the UniProt Q15717 sequence; residues outside this range were not present in the deposited coordinates and were not modeled.

### 4.3 Setup for Molecular Dynamics Simulations

Each system was inserted in a water box (TIP3P model [33]) of 20 Å isometric dimension; given the high flexibility of the protein, this allows it to freely explore its conformational space. Moreover, Na^+^ and Cl^−^ ions were added to neutralize the system at a concentration of 0.154 M. Each system was prepared using AmberTools22 [34], employing Amber14 SB [35] for the protein and OL3 [36, 37] for RNA.

Each system was subjected to a two‐step equilibration process. The first stage consisted of a 1 ns simulation in the canonical ensemble (NVT), applying harmonic positional restraints on the protein and on the nucleic acid when present. The second equilibration (isothermal–isobaric ensemble (NPT)) also consisted of 1 ns of simulation, with restraints applied only on the protein backbone and on the nucleic acid backbone when present. In all the equilibration stages, the restraint was set at 5 kcal/mol·Å^2^.

The simulations were conducted exploiting the ACEMD 3.5 engine [38]with a timestep of 2 fs. The temperature was kept constant at 310 K by means of a Langevin thermostat [39], and the pressure was kept at 1 atm by a Monte Carlo barostat [40] during the NPT phase. A switch distance of 7.5 Å for van der Waals interactions and a cutoff distance of 9 Å for van der Waals and real‐space electrostatic interactions were employed, and particle‐mesh Ewald [41] summation was used for long‐range electrostatic interactions. The ACEMD3 engine is based upon the open‐source Python library for biomolecular simulations, OpenMM [42]

### 4.4 SuMD

After equilibration, [23] SuMD was performed to obtain a simulated reclosure process of HuR. SuMD does not introduce an explicit biasing force or potential; however, its trajectory selection procedure produces a directionally selected, non‐equilibrium pathway, and the resulting trajectory cannot be used to infer equilibrium populations, kinetics, or free‐energy differences. It is an enhanced sampling MD technique originally designed to simulate recognition pathways between two distinct biological entities on a nanosecond timescale, significantly reducing the simulation time and computational costs compared to classical MD. This tool is highly versatile and has been applied to a variety of interacting partners, including ligand‐protein, RNA–protein, and protein–peptide complexes. In this case, it was employed to supervise the approach of two structural domains of the protein by monitoring the distance between their centers of mass.

SuMD operates through a series of short classical MD simulations, called SuMD steps. The distance between the centers of mass of the interacting partners (dcmL‐R) is tracked, and, at the end of each SuMD step, the distances are fitted by a linear function. If the slope of the line is negative (i.e., the two domains are approaching each other), the step is accepted and added to the final trajectory, and a new SuMD step is started from the end of the previous one. Conversely, if the slope of the line is positive (i.e., the domains are moving away from each other), the step is discarded and repeated starting from the final coordinates of the previous SuMD step, with reassignment of velocities consistent with the NVT ensemble.

The duration of the SuMD step was set to 250,000 steps with a time step of 2 fs (resulting in a 500 ps SuMD step), and the maximum number of SuMD step repetitions before SuMD stops was set to 150. Independent replicas of the same system—the three classical MD replicates and the four SuMD runs of each supervision step—differ exclusively in the random seed used to assign the initial atomic velocities from a Maxwell–Boltzmann distribution at the target temperature; all other parameters and starting coordinates were identical. Four runs per supervision step were collected, in line with previous SuMD studies, with the aim of probing the reproducibility of the supervised transition rather than of exhaustively sampling the conformational space; because SuMD steps are accepted or rejected stochastically, convergence cannot be assessed from a free‐energy estimate and was therefore evaluated in terms of the reproducibility of the final RRM2 arrangement and of the supervised distances across independent runs.

Since linker rearrangement appears to be required for closure, a single SuMD run was insufficient to reproduce the complete transition. For this reason, two consecutive runs were conducted, supervising two pairs of amino acids chosen by ranking the change in all pairwise distances between the open and the closed conformations for each simulation.

### 4.5 SuMD and Classical MD Trajectory Analysis

The analysis of SuMD and classical MD trajectories was performed using custom Tcl script and Python 3, using the MDAnalysis Python library [43, 44] and Matplotlib [45]. The RMSD and Cα‐RMSF values were calculated using VMD (version 1.9.3) using the default backbone definition of the RMSD Trajectory Tool (atoms N‐Cα‐C) for the RMSD analysis. Per‐residue RMSD analysis were computed with a Python script employing MDAnalysis functionalities. Energetic profiling involved estimating ligand–receptor interaction energies throughout the trajectory using the NAMD Energy plugin based on the NAMD engine [46] and the AMBER14 force field. Interaction energies were calculated as the sum of van der Waals and electrostatic contributions. Additionally, the per‐residue decomposition of the interaction energy, generated through the same plugin, adds the time‐resolved energy contributions from protein amino acids and nucleotides of the nucleic acid ligand. The most frequently contacting residues from each component were considered, with contacts defined as atom pairs within a maximum distance of 4.5 Å. For consistency, all residue numbering shown in plots follows the canonical UniProt [47] FASTA sequence of the HuR protein Q15717. A video was generated by employing VMD for structure rendering and Matplotlib for plot generation.

### 4.6 TTClust

TTClust [29] was used to cluster molecular dynamics simulation trajectories. It is a Python program able to process the trajectories of major simulation programs (e.g., Gromacs, AMBER, CHARMM, and NAMD and any trajectories in the PDB format), thanks to the MDTraj package. In this work, the clustering was performed using the default autoclustering procedure, which estimates the optimal number of clusters using the elbow method and applies Ward’s hierarchical linkage. Specifically, each frame was structurally aligned using the user‐defined alignment selection, and a pairwise RMSD distance matrix was then calculated using the user‐defined RMSD selection. For the global analysis, the alignment was performed on the residues of RRM1 domain (Gly18‐Ala96), and the RMSD was calculated on all the protein backbone atoms. This matrix was then used to generate a hierarchical dendrogram through Ward’s linkage, which progressively merges clusters while minimizing the increase in within‐cluster variance. The dendrogram illustrates the successive merging of conformational clusters, with the height of each node representing the corresponding intercluster distance. The autoclustering procedure was then applied to identify a suitable number of clusters by evaluating the clustering profile and selecting the elbow point, beyond which the addition of further clusters resulted in only a limited improvement in cluster compactness. For each cluster, TTClust identified a representative frame as the structure with the lowest mean pairwise RMSD to all other frames belonging to that cluster.

### 4.7 Fpocket

Binding pockets on the HuR SuMD reclosure trajectory were identified using fpocket [30, 31] (version 4.0). fpocket is a geometry‐based pocket detection algorithm that relies on Voronoi tessellation and α‐sphere theory to delineate cavities on the protein surface.

In brief, the three‐dimensional space around the protein is decomposed into Voronoi cells, from which α‐spheres are derived. An α‐sphere is defined as a sphere that touches four protein atoms on its boundary without enclosing any atom within its volume. Spheres that are too small (reflecting tightly packed regions located within the protein) or too large (corresponding to solvent‐exposed areas) are filtered out, retaining only those of intermediate size that are indicative of potential binding pockets. The filtering criteria are based on minimal (default value: 3 Å) and maximal (default value: 6 Å) radii values. The remaining α‐spheres were then clustered based on spatial proximity to define candidate pockets. Each pocket is characterized by a set of geometric and physicochemical descriptors, including volume, solvent‐accessible surface area, hydrophobicity, polarity, charge, and α‐sphere density. Pockets are subsequently ranked using a druggability score (from 0–1, the higher the better), computed via a partial least squares regression model trained on known binding sites, which estimates the likelihood that a given pocket can accommodate drug‐like small molecules.

fpocket was run on the resulting structures obtained after clustering the SuMD reclosure pathway trajectory using the program in its default parameters. The top‐ranked pockets according to the druggability score ≥0.5 were retained for further analysis. Structural visualization and mapping of pocket residues were performed using VMD.

## Funding

This work was supported by the European Union ‐ Next‐Generation EU (PNRR M4C2‐Investimento 1.4‐CN00000041).

## Conflicts of Interest

The authors declare no conflicts of interest.

## Supporting information

Supplementary Material

Supplementary Material

## Data Availability

The code to perform SuMD simulations is available free of charge at https://github.com/molecularmodelingsection/SuMD.
