# Supplementary material for: Capturing Hu Antigen R Domain Closure Through Supervised Molecular Dynamics Simulations
Source: ChemMedChem. 2026 Oct 4;21(19):e70523. doi: 10.1002/cmdc.70523 (PMC13635762; doi:10.1002/cmdc.70523)
Supplement: Supplementary file 1 [file CMDC-21-e70523-s001.pdf]

Supporting Information

for

Capturing HuR Domain Closure through Supervised Molecular Dynamics Simulations

Chiara Cavastracci Strascia,[1] Andrea Dodaro,[1] Gianluca Novello,[1] Silvia Menin,[1] Mattia Sturlese,[1] Veronica Salmaso,[1] Stefano Moro,*[1]

[1] Molecular Modeling Section (MMS), Department of Pharmaceutical and Pharmacological Sciences, University of Padova, via Marzolo 5, 35131 Padova, Italy

Address correspondence to stefano.moro@unipd.it

Table of contents

Table S1. RMSD of RRM1 and RRM2 in Apo, RNA-Bound, and Pseudo-Apo States 5

Table S2. Top-ranked residue-pair distances and Δ-distances. 8

Figure S1. RMSD profiles of RRM1 and RRM2 during three 500 ns MD simulations of apo, RNA-bound and pseudo-apo states. 3

Figure S2. RMSF profiles of RRM1 and RRM2 during three 500 ns MD simulations of apo, RNA-bound and pseudo-apo states. 4

Figure S3. RMSD of RRM2 from the X-ray closed conformation during apo MD simulations. 6

Figure S4. RMSD of RRM2 from the X-ray closed conformation during MD simulations of the RNA-bound apo model. 7

Figure S5. RMSD of RRM2 from the X-ray closed conformation during Productive SuMD simulations 9

Figure S6. HuR-RNA interaction energy during SuMD simulations. 10

Figure S7. Per-residue RMSD analysis of the final state of the best SuMD run as compared to the X-ray closed structure. 11

Figure S8. RMSD of RRM2 from the X-ray closed conformation of the last frame of the best Productive SuMD simulation during 100 ns MD. 12

Figure S9. Last frames of Multi-step Productive SuMD simulations. 13

Figure S10. Last frames of Multi-step Unproductive SuMD simulations. 14

Video S1 18

Figure S1

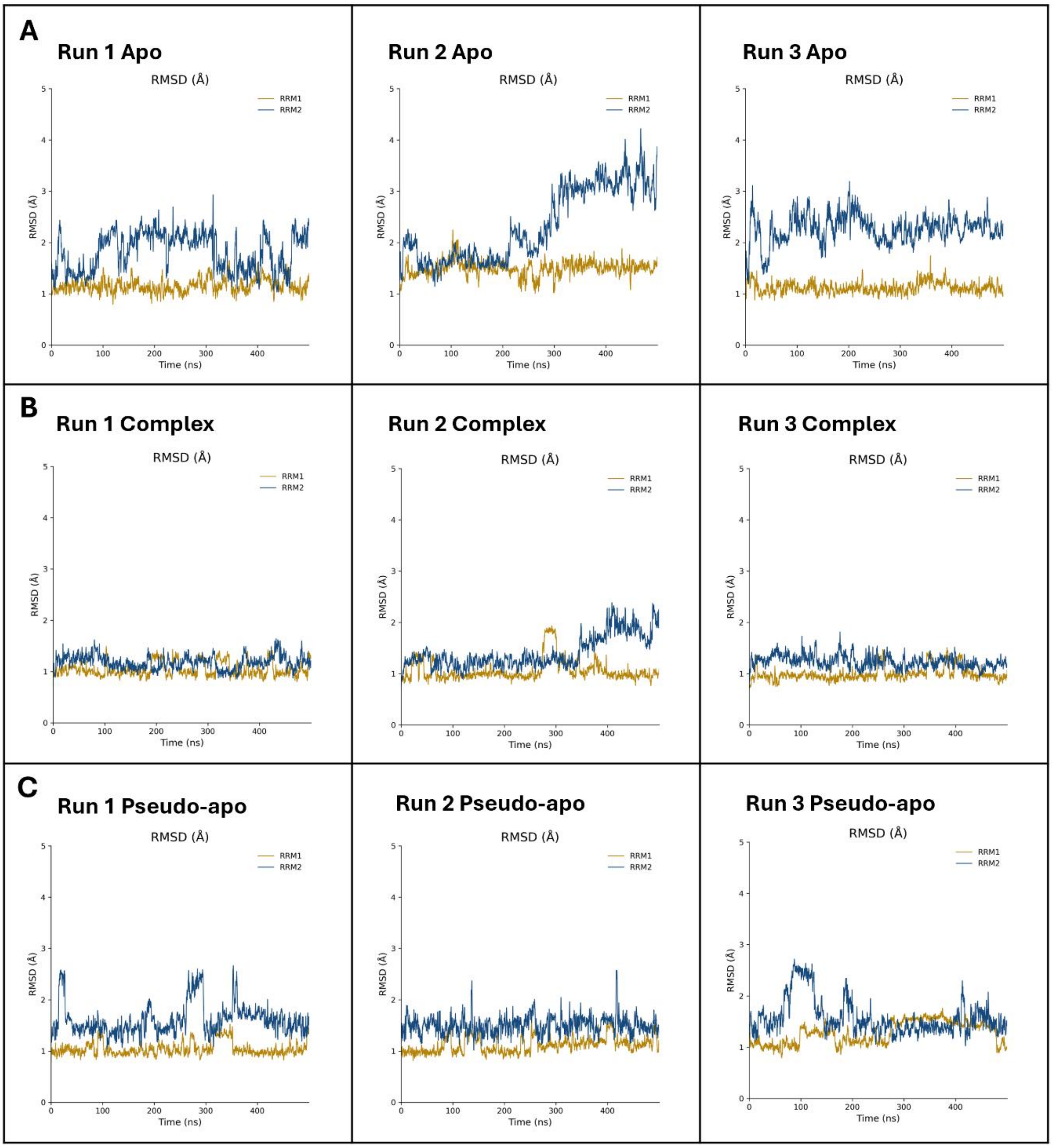

Figure S1. RMSD profiles of RRM1 and RRM2 during three 500 ns MD simulations of apo, RNA-bound and pseudo-apo states. Backbone RMSD profiles of RRM1 (gold) and RRM2 (blue) from three independent 500 ns MD simulations for each of the apo (A), RNA-bound (B), and pseudo-apo (C) states. For each domain, RMSD was calculated using its own initial conformation (the X-ray state) as the reference structure, after structural alignment to the same domain.

Figure S2

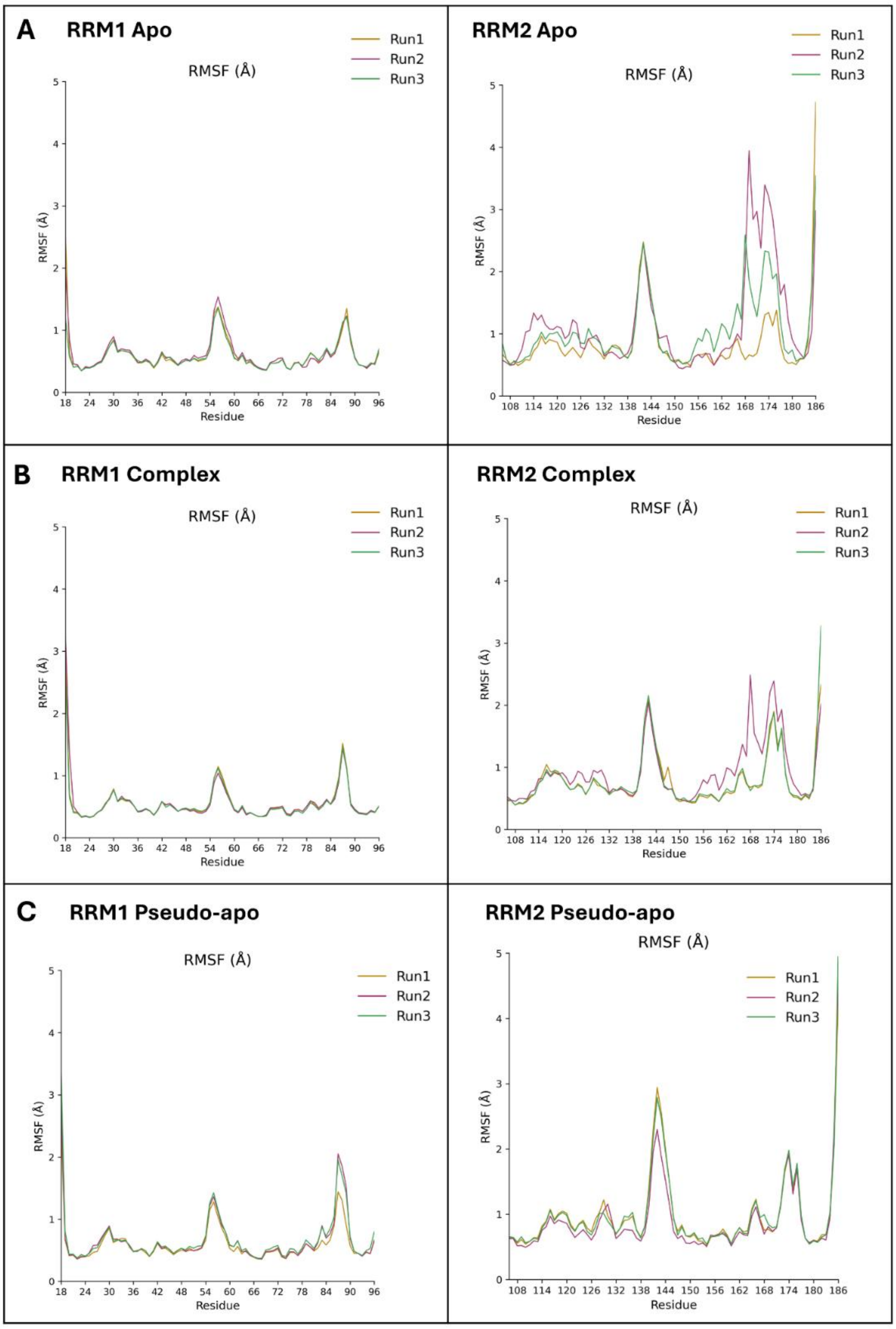

Figure S2. RMSF profiles of RRM1 and RRM2 during three 500 ns MD simulations of apo, RNA-bound and pseudo-apo states. Cα-RMSF profiles of RRM1 and RRM2 from three independent 500 ns MD simulations for each of the apo (A), RNA-bound (B), and pseudo-apo (C) states. For each domain, per-residue fluctuations were computed with respect to the average structure of the trajectory, after least-squares superposition of that domain onto its own initial (X-ray) conformation.

Table S1

Domain	Mean RMSD	Standard Deviation	Inter-replicate mean RMSD	Standard Deviation (all data)
RRM1 Apo	Run 1: 1.16 Å	0.17	1.26 Å	2.41
	Run 2: 1.49 Å	0.19		
	Run 3: 1.22 Å	0.16		
RRM2 Apo	Run 1: 1.82 Å	0.40	2.14 Å	0.56
	Run 2: 2.33 Å	0.73		
	Run 3: 2.26 Å	0.31		
RRM1 Complex	Run 1: 1.04 Å	0.16	1.03 Å	0.19
	Run 2: 1.06 Å	0.23		
	Run 3: 1 Å	0.16		
RRM2 Complex	Run 1: 1.18 Å	0.18	1.28 Å	0.26
	Run 2: 1.41 Å	0.34		
	Run 3: 1.24 Å	0.17		
RRM1 Pseudo-apo	Run 1: 1.04 Å	0.15	0.15 Å	0.22
	Run 2: 1.12 Å	0.18		
	Run 3: 1.28 Å	0.24		
RRM2 Pseudo-apo	Run 1: 1.61 Å	0.33	1.56 Å	0.32
	Run 2: 1.50 Å	0.23		
	Run 3: 1.58 Å	0.38		

Table S1. RMSD of RRM1 and RRM2 in Apo, RNA-Bound, and Pseudo-Apo States. Mean RRM1 and RRM2 backbone RMSD values in the apo, RNA-bound and pseudo-apo states across three independent MD replicates. For each condition, the table reports the average RMSD of each run and the overall inter-replicate mean. RMSD was computed separately for RRM1 and RRM2 by aligning each domain onto its corresponding reference structure. Standard deviations are also reported for each mean value, with the overall standard deviation calculated across all data points from the three replicates.

Figure S3

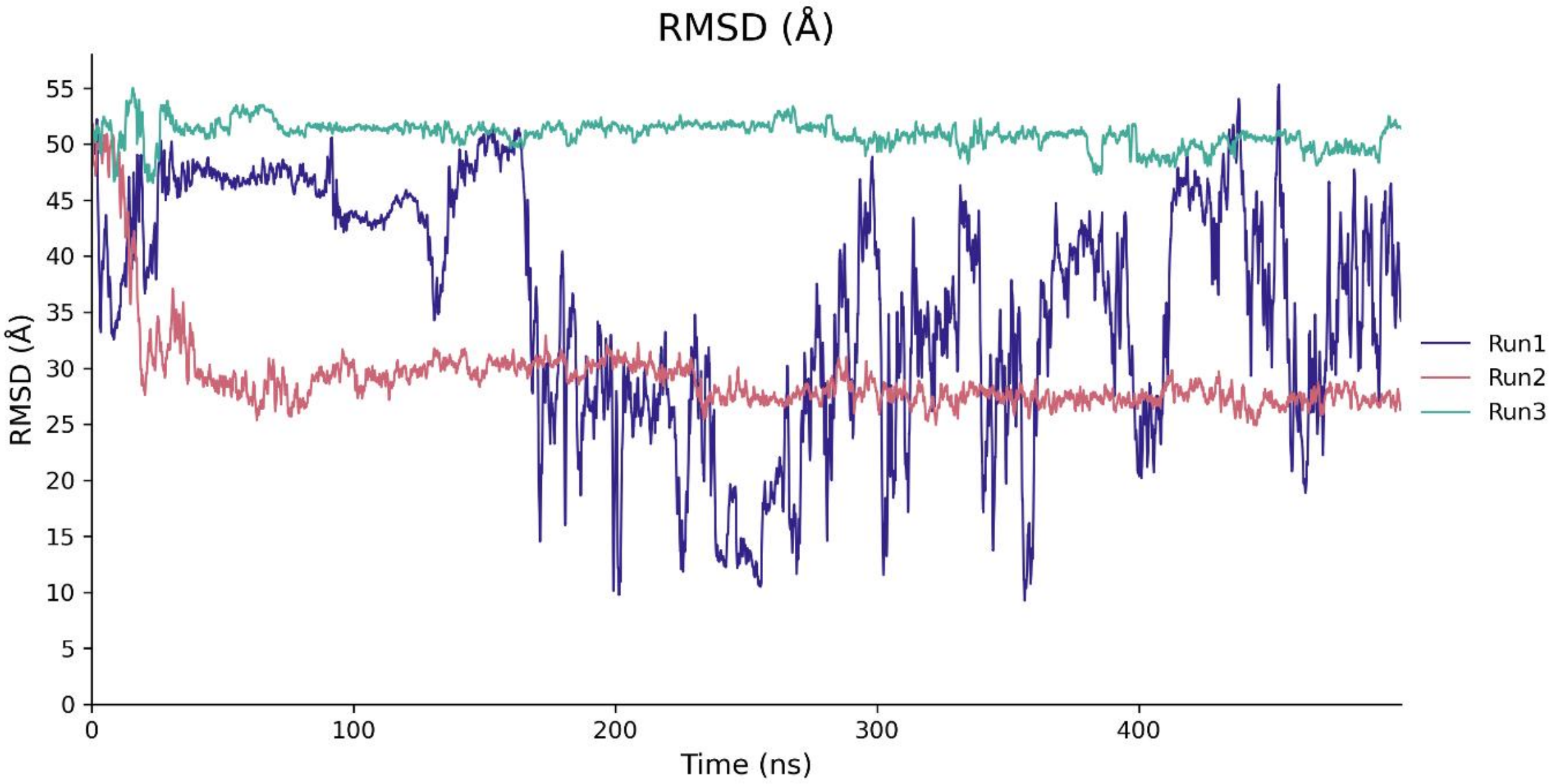

Figure S3. RMSD of RRM2 from the X-ray closed conformation during apo MD simulations. Backbone RMSD of RRM2 of the apo structure (PDB ID: 4EGL) relative to RRM2 of the X-ray closed conformation (PDB ID: 4ED5) during three independent 500 ns MD simulations. RMSD values were calculated after structural alignment to RRM1 backbone of the closed X-ray structure. Note that this metric, computed after superposition on RRM1, intentionally combines the relative movement of the two domains and any internal deformation of RRM2; the two contributions are separated in Figure S7.

Figure S4

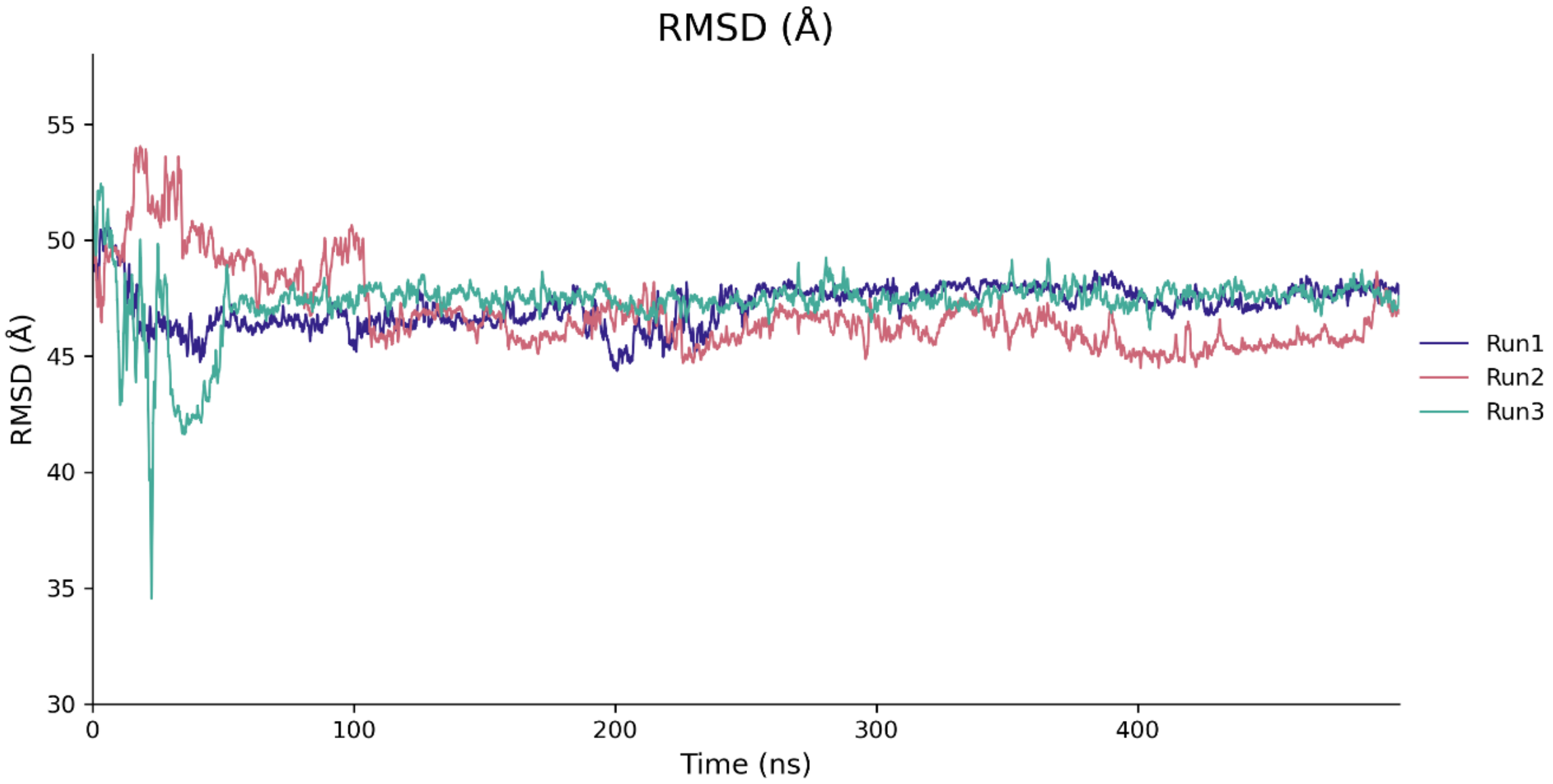

Figure S4. **RMSD of RRM2 from the X-ray closed conformation during MD simulations of the RNA-bound apo model.** Backbone RMSD of RRM2 of the RNA-bound apo model relative to RRM2 of the closed X-ray structure (PDB ID: 4ED5) during three independent 500 ns MD simulations. RMSD values were calculated after structural alignment to RRM1 backbone of the closed X-ray structure. The RNA-bound apo model was built by superposing RRM1 of the RNA-bound closed X-ray structure (PDB ID: 4ED5) onto RRM1 of the apo structure (PDB ID: 4EGL) and transferring the RNA accordingly, so that the RNA is engaged by RRM1 only while the two domains retain the open arrangement of the apo form.

Table S2

Residue1	Residue2	Distance in Apo Open State	Distance in RNA-bound Closed State	Delta Distance
Leu 61	Pro 186	31.48 Å	12.30 Å	19.18 Å
Lys92	Asn134	26.45 Å	10.99 Å	15.46 Å
Ile103	Lys156	13.01 Å	4.46 Å	8.56 Å

Table S2. Top-ranked residue-pair distances and Δ-distances. Distance values of the three top-ranked residue pairs in the apo open state, in RNA-bound closed state and the corresponding Δ-distance values, calculated as the difference between the open- and closed-state distances.

Figure S5

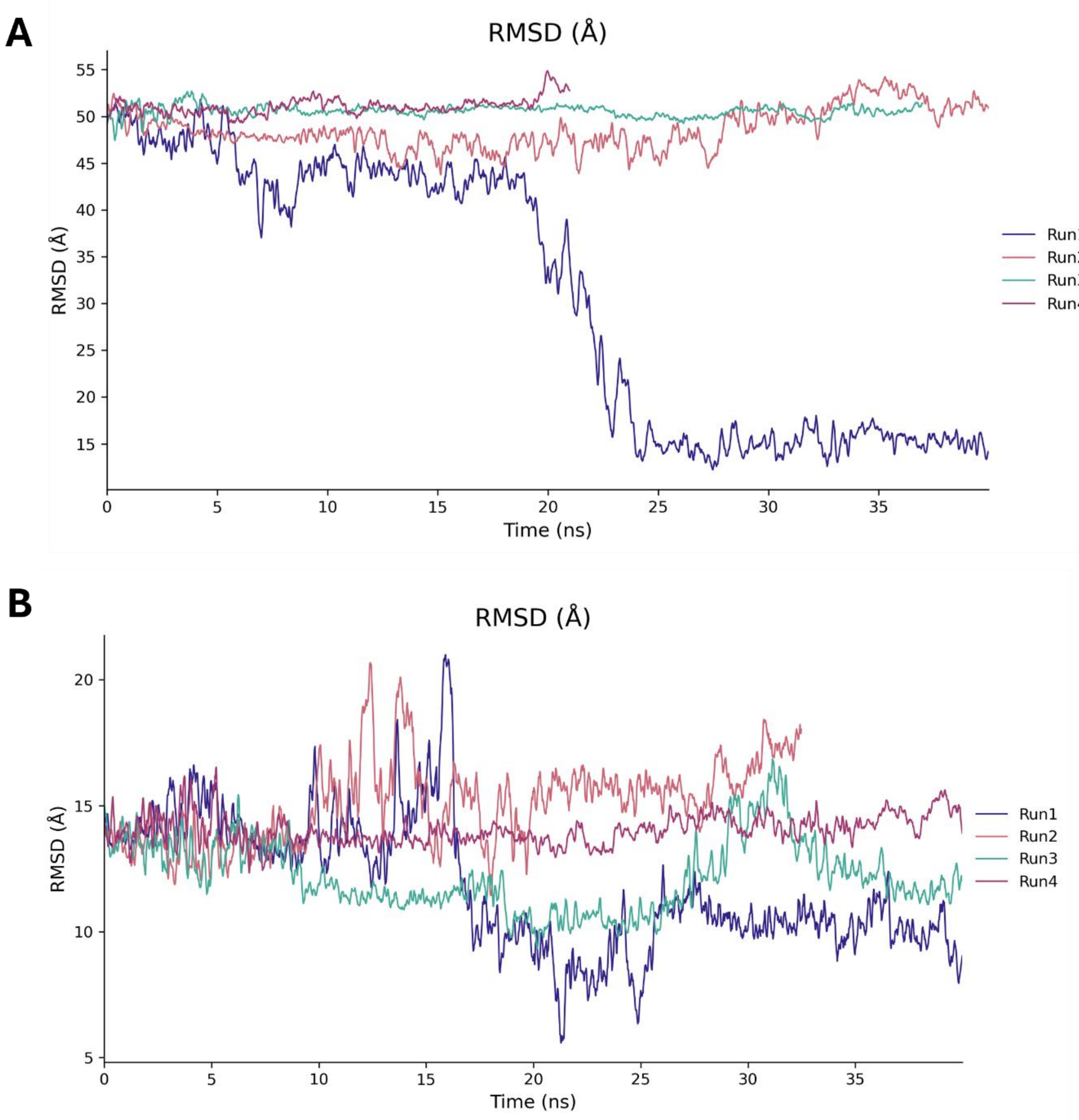

Figure S5. **RMSD of RRM2 from the X-ray closed conformation during Productive SuMD simulations**. Backbone RMSD of RRM2 of RNA-bound apo model relative to RRM2 of the closed X-ray structure (PDB ID: 4ED5) during Supervised Molecular Dynamics (SuMD) simulations. RMSD values were calculated after structural alignment to RRM1 backbone of the closed X-ray structure. The first panel (A) reports the RMSD profiles from 4 Productive Supervision Step-1 runs; and the second (B) reports RMSD profiles from 4 Productive Supervision Step-2 runs. Here, “Productive” refers to the order in which the two supervised residue pairs are imposed (Ile103–Lys156 first, followed by Lys92–Asn134), and not to the outcome of the simulation: among the Supervision Step-1 replicas, only run 1 approaches the closed conformation, whereas runs 2–4 remain close to the initial open state. All Supervision Step-2 replicas were seeded from the final frame of the productive Supervision Step-1 run (run 1).

Figure S6

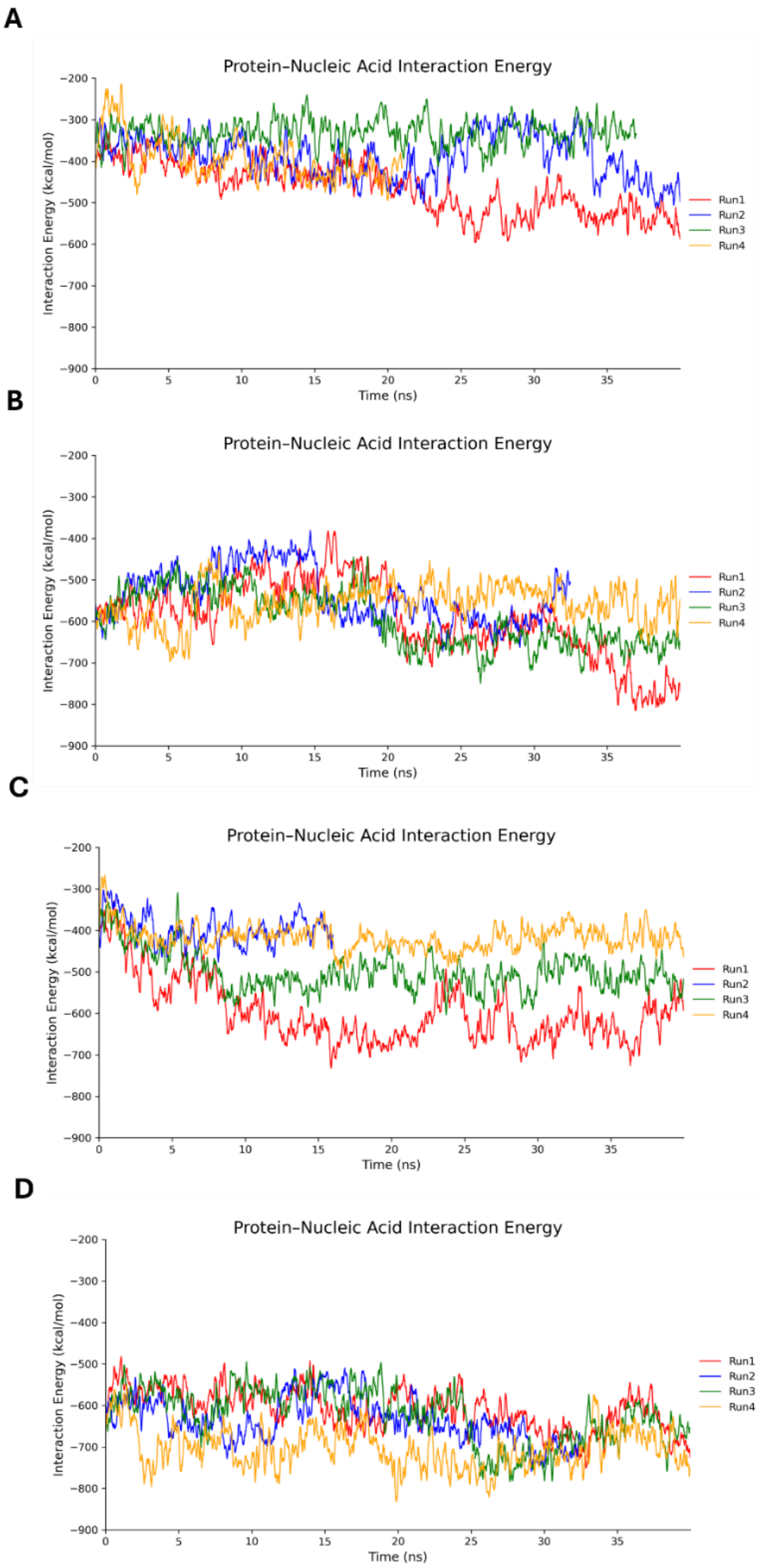

Figure S6. HuR-RNA interaction energy during SuMD simulations. Interaction energy between HuR and RNA during SuMD simulations. **A.** Interaction energy profile from 4 Productive Supervision Step-1 runs; **B.** Interaction energy profile from 4 Productive Supervision Step-2 runs. **C.** Interaction energy profile from 4 Unproductive Supervision Step-1 runs; **D.** Interaction energy profile from 4 Unproductive Supervision Step-2 runs. Interaction energies were computed as the sum of the van der Waals and electrostatic non-bonded terms between HuR and the RNA with the NAMD Energy plugin, using [cut-off] and [dielectric treatment; solvent and counter-ions included/excluded]. These are raw non-bonded interaction energies and are not to be interpreted as binding free energies; they are used here only as a relative, qualitative descriptor of RNA engagement. Comparable values are reached under the Productive and the Unproductive protocols, indicating that the RNA is engaged in both cases and that it is the RMSD of RRM2 from the closed conformation, rather than the interaction energy, that discriminates productive from unproductive domain closure.

Figure S7

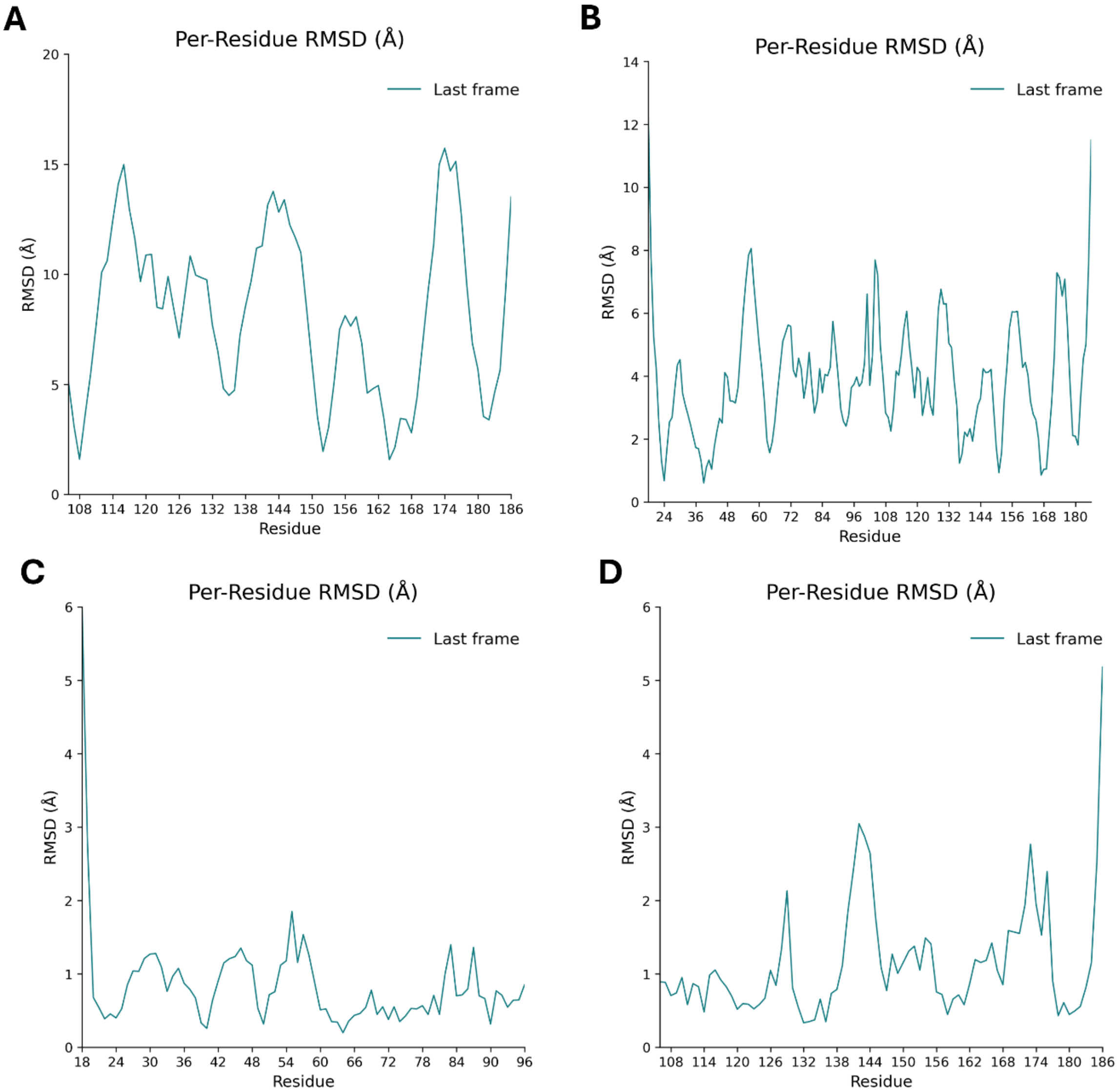

Figure S7. Per-residue RMSD analysis of the final state of the best SuMD run as compared to the X-ray closed structure. All the per-residue RMSD profiles were computed by comparing each residue in the last frame of the best Productive SuMD simulation (run1, Supervision Step-2) with the corresponding residue in the reference RNA-bound closed structure (PDB ID: 4ED5). The different RMSD profiles were generated after aligning the protein to different portions of the reference, as follows. (A) Per-residue backbone RMSD of RRM2 calculated after alignment to RRM1 (overall RRM2 RMSD of 9.04 Å). (B) Per-residue backbone RMSD of the entire protein after alignment to the whole backbone (overall backbone RMSD of 4.33 Å). (C) Per-residue backbone RMSD of RRM1 calculated after alignment to RRM1 (overall RRM1 RMSD of 1.19 Å). (D) Per-residue backbone RMSD of RRM2 calculated after alignment to RRM2 (overall RRM2 RMSD of 1.38 Å). Per-residue values are defined as the deviation of the backbone atoms (N, Cα, C, O) of each residue between the two superimposed structures. Each panel uses a different superposition in order to separate the contributions to the overall deviation, namely the rigid-body displacement of RRM2 relative to RRM1 (A) from the internal deformation of each individual domain (C, D). The low intra-domain values obtained for RRM1 (1.19 Å) and RRM2 (1.38 Å) indicate that the deviation reported in panel A is dominated by the relative movement of the two domains rather than by intra-domain distortion.

Figure S8

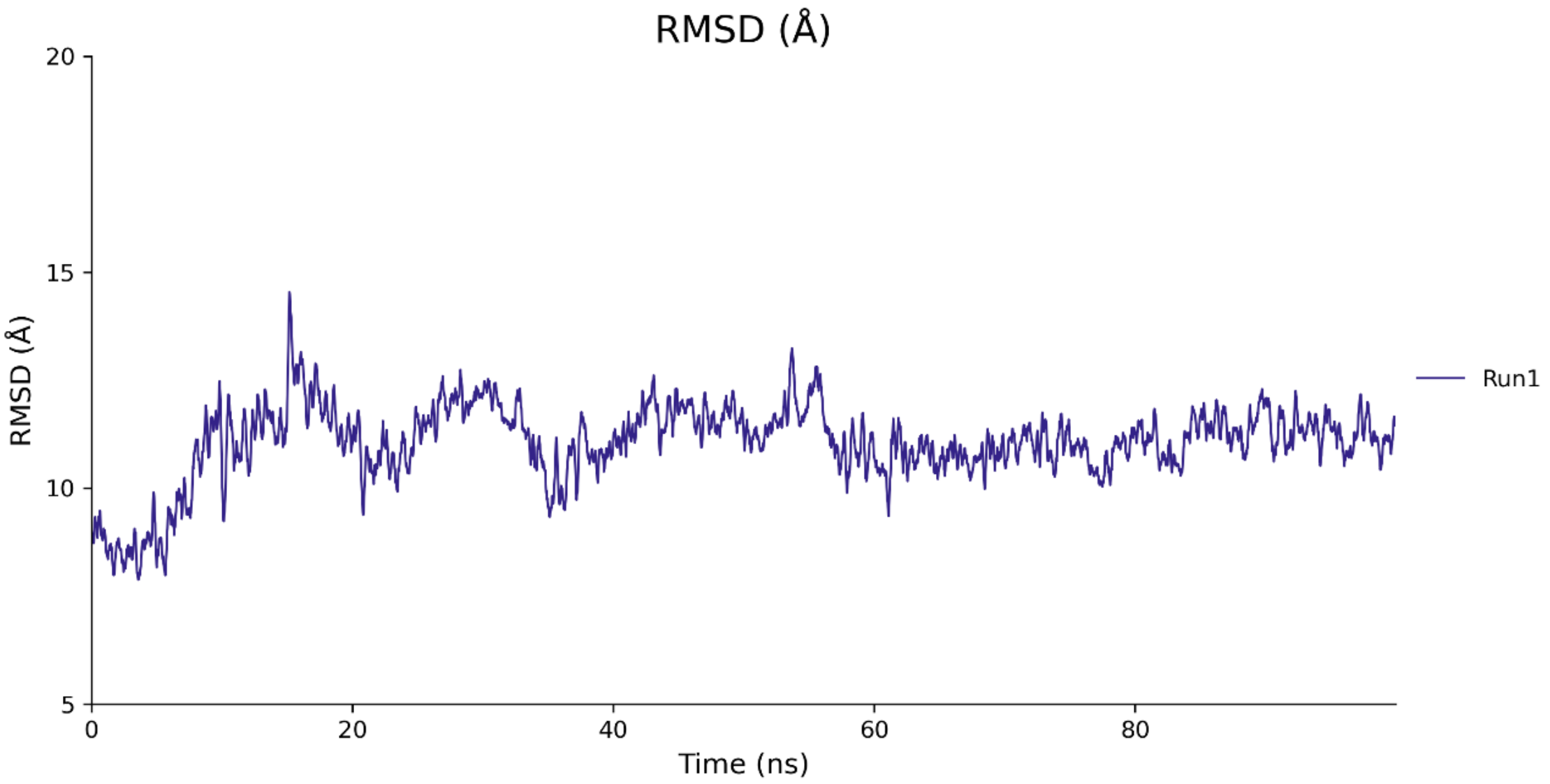

Figure S8. **RMSD of RRM2 from the X-ray closed conformation of the last frame of the best Productive SuMD simulation during 100 ns MD.** Backbone RMSD profile of RRM2 of the last frame of Productive SuMD simulation (run 1, Supervision Step-2) compared to RRM2 of the closed X-ray reference structure (PDB ID: 4ED5) during a 100 ns MD simulation. RMSD values were calculated after structural alignment to RRM1 backbone of the closed X-ray structure. A single replica is reported. Over the 100 ns the RMSD does not decrease further but slowly drifts from approximately 9 to 11 Å, indicating that the end-state of the SuMD simulation is metastable on this timescale rather than relaxing towards the crystallographic closed conformation.

Figure S9

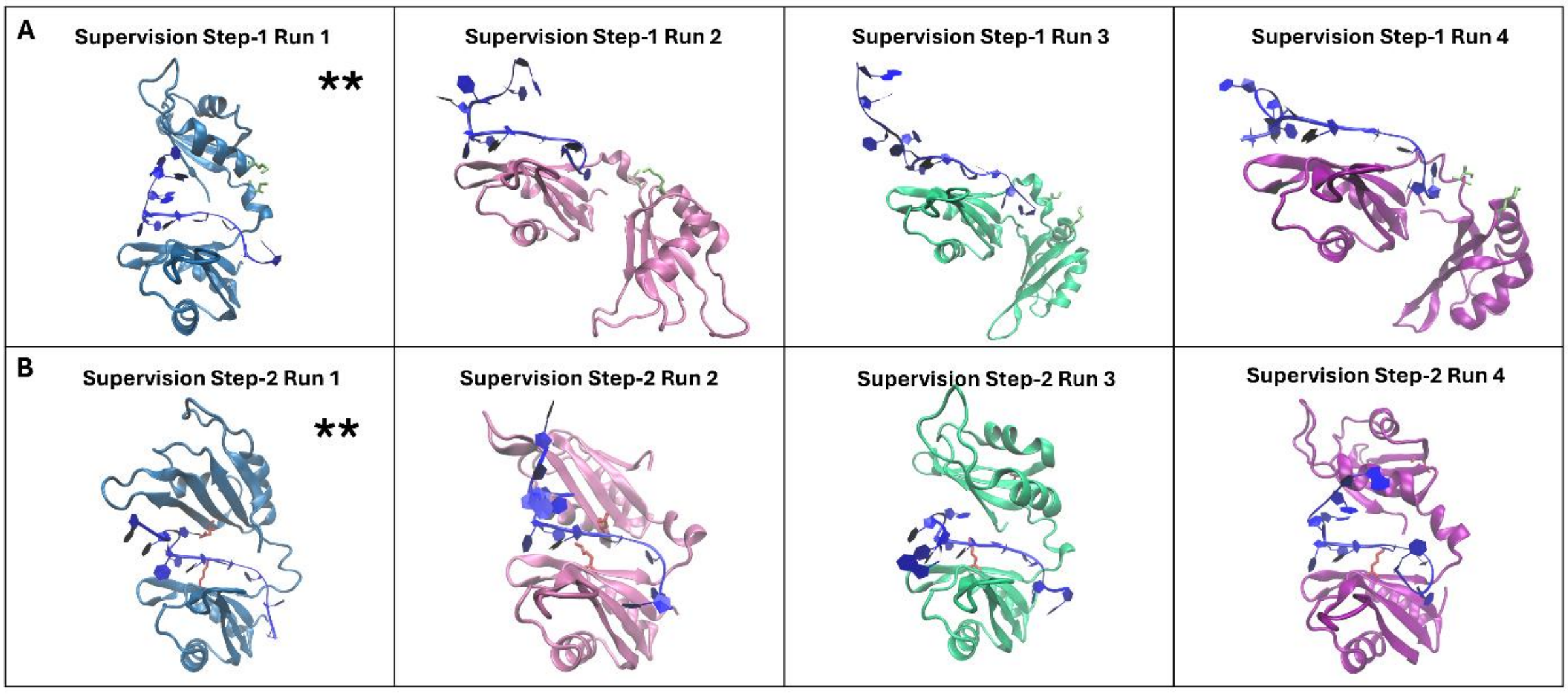

Figure S9. Last frames of Multi-step Productive SuMD simulations. Representation of last frames obtained after Supervision Step-1 (A) and Supervision Step-2 of Multi-step Productive SuMD simulations (B). For each complex, RNA is coloured dark blue, whereas the protein is coloured light blue, pink, green, and purple in run1, run2, run3, and run4, respectively. Moreover, the final position of the residue pairs used for supervision are shown (Ile103 and Lys156, coloured in lime, in Supervision Step-1; Lys92 and Asn134, coloured in red, in Supervision Step-2). The selected simulations are highlighted by **.

Figure S10

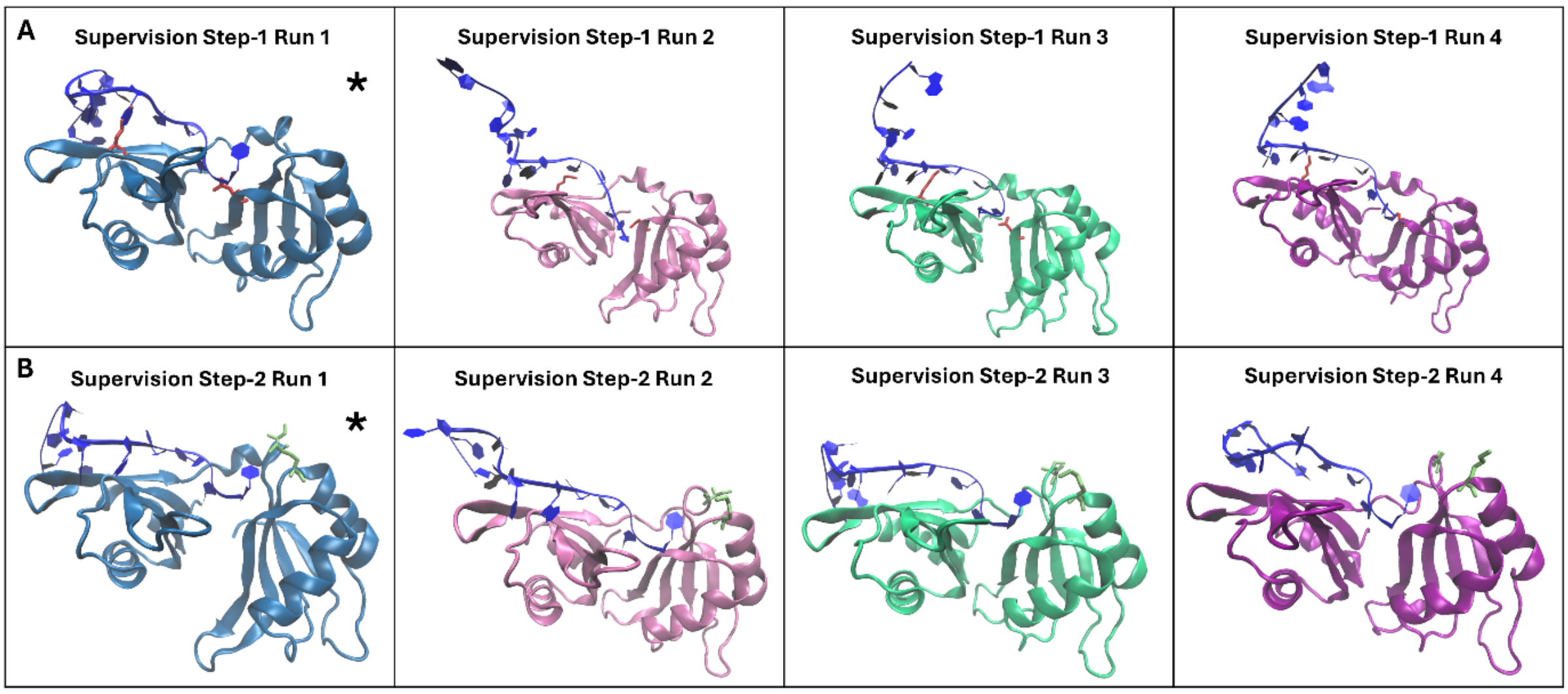

Figure S10. Last frames of Multi-step Unproductive SuMD simulations. Representation of last frames obtained after Supervision Step-1 (A) and Supervision Step-2 (B) of Multi-step Unproductive SuMD simulations. For each complex, RNA is coloured dark blue, whereas the protein is coloured light blue, pink, green, and purple in run1, run2, run3, and run4, respectively. Moreover, the final position of the residue pairs used for supervision are shown (Lys92 and Asn134 in Supervision Step-1; Ile103 and Lys156 in Supervision Step-2). The selected simulations are highlighted by *.

Video S1

Video S1 illustrates the SuMD reclosure process together with the protein–nucleic acid interaction-energy profile and RMSD of RRM2 along time. In particular, the left panel depicts the trajectory, where RNA is reported in dark blue, RRM1 in yellow, the linker in red, RRM2 in light blue, and the reference closed X-ray structure in transparent grey. The right panel consists of a scatter plot reporting RRM2 RMSD on the y-axis, time on the x-axis, while the protein-RNA interaction energy is rendered by a colorimetric scale (in which darker blue corresponds to more negative energy values). The video highlights the progressive movement of RRM2 toward the nucleic acid during re-closure. As the system approaches the closed topology, the interaction-energy values become progressively more favorable.
